# Supplementary material for: Jordan products of quantum channels and their compatibility
Source: Nat Commun. 2021 Apr 9;12:2129. doi: 10.1038/s41467-021-22275-0 (PMC8035191; doi:10.1038/s41467-021-22275-0)
Supplement: Supplementary file 1 — Supplementary Information [file 41467_2021_22275_MOESM1_ESM.pdf]

Supplementary Information for "Jordan products of quantum channels and their compatibility"

Mark Girard [1] Martin Plávala [2, 3*] Jamie Sikora [4, 5, 1]

Supplementary Note 1 - Notation and background

In this section we summarize the terminology and notation used in this paper and review some of the basic known facts and results concerning compatibility in quantum theory. Note that we mostly use the same notation as in [1].

1.1 Notation

In this paper we work with (finite-dimensional) complex Euclidean spaces, which we may assume to be of the form $\mathbb{C}^n$ for some positive integer n. We reserve the notation $\mathcal{X}, \mathcal{X}_1, \ldots, \mathcal{X}_n$, $\mathcal{Y}, \mathcal{Y}_1, \ldots, \mathcal{Y}_n$ for complex Euclidean spaces. We use the following notation for frequently used sets of linear operators and linear maps of operators.

- $\mathrm{L}(\mathcal{X}, \mathcal{Y})$ is the space of linear operators from $\mathcal{X}$ to $\mathcal{Y}$, and we write $\mathrm{L}(\mathcal{X})$ when $\mathcal{X} = \mathcal{Y}$.
- $\mathrm{U}(\mathcal{X}, \mathcal{Y})$ is the set of isometries from $\mathcal{X}$ to $\mathcal{Y}$, while $\mathrm{U}(\mathcal{X})$ is the set of unitaries acting on $\mathcal{X}$.
- $\mathrm{Herm}(\mathcal{X})$ is the set of Hermitian operators acting on $\mathcal{X}$.
- $\mathrm{Pos}(\mathcal{X})$ is the set of Hermitian, positive semidefinite operators acting on $\mathcal{X}$.
- $\mathrm{D}(\mathcal{X})$ is the set of density matrices acting on $\mathcal{X}$.
- $\mathrm{T}(\mathcal{X}, \mathcal{Y})$ is the set of linear maps from $\mathrm{L}(\mathcal{X})$ to $\mathrm{L}(\mathcal{Y})$, and we write $\mathrm{T}(\mathcal{X})$ when $\mathcal{X} = \mathcal{Y}$.
- $\mathrm{C}(\mathcal{X}, \mathcal{Y})$ is the set of quantum channels from $\mathcal{X}$ to $\mathcal{Y}$, and we write $\mathrm{C}(\mathcal{X})$ when $\mathcal{X} = \mathcal{Y}$.
- $\mathrm{Sep}(\mathcal{X} : \mathcal{Y})$ is the set of separable operators acting on $\mathcal{X} \otimes \mathcal{Y}$.
- $\mathrm{PPT}(\mathcal{X} : \mathcal{Y})$ is the set of PPT operators acting on $\mathcal{X} \otimes \mathcal{Y}$. (An operator $X \in \mathrm{L}(\mathcal{X} \otimes \mathcal{Y})$ is said to be PPT (positive partial transpose) if both X and its partial transpose $X^{T_\mathcal{X}}$ are positive semidefinite.)

[1]Institute for Quantum Computing, University of Waterloo, Ontario, Canada. mark.girard@uwaterloo.ca
[2]Naturwissenschaftlich-Technische Fakultät Universität Siegen, Siegen, Germany. martin.plavala@uni-siegen.de
[3]Mathematical Institute, Slovak Academy of Sciences, Bratislava, Slovakia.
[4]Virginia Polytechnic Institute and State University, Blacksburg, Virginia, USA. sikora@vt.edu
[5]Institute for Quantum Computing, University of Waterloo, Waterloo, Ontario, Canada.

Every complex Euclidean space $\mathcal{X} = \mathbb{C}^n$ comes equipped with an inner product defined as $\langle x, y \rangle = \sum_{i=1}^{n} \overline{x_i} y_i$ for every $x, y \in \mathcal{X}$. For an operator $A \in \mathrm{L}(\mathcal{X}, \mathcal{Y})$, its adjoint $A^* \in \mathrm{L}(\mathcal{Y}, \mathcal{X})$ is the unique operator that satisfies $\langle Ax, y \rangle = \langle x, A^* y \rangle$ for every $x \in \mathcal{X}$ and $y \in \mathcal{Y}$. The space $\mathrm{L}(\mathcal{X}, \mathcal{Y})$ is itself a complex Euclidean space with Hilbert–Schmidt inner product given by

$$\langle A, B \rangle = \mathrm{Tr}(A^* B) \tag{1}$$

for every $A, B \in \mathrm{L}(\mathcal{X}, \mathcal{Y})$. A self-adjoint operator $\Pi \in \mathrm{L}(\mathcal{X})$ is a projection operator if it satisfies $\Pi^2 = \Pi$. For a subspace $\mathcal{V} \subseteq \mathcal{X}$, we denote the projection operator onto the subspace $\mathcal{V}$ as the operator $\Pi_{\mathcal{V}}$. For an operator $X \in \mathrm{L}(\mathcal{X})$ we denote the image of X as the subspace

$$\mathrm{im}(X) = \{Xu : u \in \mathcal{X}\} \subseteq \mathcal{X}. \tag{2}$$

We use the notation $A \geq B$ to mean that the operator $A - B$ is positive semidefinite. The identity operator (i.e., identity matrix) on $\mathcal{X}$ is denoted $\mathbb{1}_{\mathcal{X}}$.

A quantum channel is a completely positive and trace-preserving linear map $\Phi \in \mathrm{T}(\mathcal{X}, \mathcal{Y})$. The identity channel on $\mathcal{X}$ is denoted $\mathbb{1}_{\mathrm{L}(\mathcal{X})}$. Quantum channels are often presented in terms of their Choi representations. For a linear map $\Phi \in \mathrm{T}(\mathcal{X}, \mathcal{Y})$, the Choi representation of Φ is the operator $J(\Phi) \in \mathrm{L}(\mathcal{X} \otimes \mathcal{Y})$ defined as

$$J(\Phi) = \sum_{i,j=1}^{\dim(\mathcal{X})} E_{i,j} \otimes \Phi(E_{i,j}). \tag{3}$$

The Choi representation satisfies the following properties:

- Φ is completely positive if and only if $J(\Phi) \geq 0$.
- Φ is trace preserving if and only if $\mathrm{Tr}_{\mathcal{Y}}(J(\Phi)) = \mathbb{1}_{\mathcal{X}}$, where $\mathrm{Tr}_{\mathcal{Y}}$ denotes the partial trace over $\mathcal{Y}$.
- Φ is entanglement-breaking if and only if $J(\Phi) \in \mathrm{Sep}(\mathcal{X} : \mathcal{Y})$.

One recovers the action of the linear map $\Phi \in \mathrm{C}(\mathcal{X}, \mathcal{Y})$ on a matrix $X \in \mathrm{L}(\mathcal{X})$ from its Choi representation by the equation

$$\Phi(X) = \mathrm{Tr}_{\mathcal{X}}\big((X^T \otimes \mathbb{1}_{\mathcal{Y}}) J(\Phi)\big), \tag{4}$$

where X^T denotes the transpose of an operator X. Note that the Choi representation of a linear map $\Phi \in \mathrm{T}(\mathcal{X}, \mathcal{Y})$ can also be defined via the equation

$$J(\Phi) = (\mathbb{1}_{\mathrm{L}(\mathcal{X})} \otimes \Phi)(J(\mathbb{1}_{\mathrm{L}(\mathcal{X})})). \tag{5}$$

For a linear map $\Phi \in \mathrm{T}(\mathcal{X}, \mathcal{Y})$, its adjoint $\Phi^* \in \mathrm{T}(\mathcal{Y}, \mathcal{X})$ is the unique linear map that satisfies $\langle \Phi(X), Y \rangle = \langle X, \Phi^*(Y) \rangle$ for every $X \in \mathrm{L}(\mathcal{X})$ and $Y \in \mathrm{L}(\mathcal{Y})$. Note that the adjoint of a partial trace map is equivalent to tensoring with an identity operator. For example, the adjoint of the map $\mathrm{Tr}_{\mathcal{Y}} \in \mathrm{T}(\mathcal{X} \otimes \mathcal{Y}, \mathcal{X})$ is given as

$$\mathrm{Tr}_{\mathcal{Y}}^*(X) = X \otimes \mathbb{1}_{\mathcal{Y}} \tag{6}$$

for every $X \in \mathrm{L}(\mathcal{X})$.

1.2 Quantum measurements and measure-and-prepare channels

A measurement in quantum theory is a procedure that assigns probabilities to quantum states. Every measurement on a complex Euclidean space $\mathcal{X}$ is described by a positive-operator valued measure (POVM)—a collection of positive semidefinite operators $\{M_i : i \in \Gamma\} \subset \mathrm{Pos}(\mathcal{X})$ for some finite set Γ of measurement outcomes that satisfies

$$\sum_{i \in \Gamma} M_i = \mathbb{1}_{\mathcal{X}}. \tag{7}$$

If the state of the system is described by a given density operator $\rho \in \mathrm{D}(\mathcal{X})$, the probability of obtaining a particular outcome $i \in \Gamma$ when measuring the system is equal to $\langle M_i, \rho \rangle$. Without loss of generality, the finite set of measurement outcomes may be assumed to be of the form $\Gamma = \{1, \ldots, m\}$ for some positive integer m.

A projective-valued measure (PVM) is a POVM of the form $\{\Pi_i : i \in \Gamma\}$ such that the operator Π_i is a projection operator for each outcome $i \in \Gamma$. One necessarily has that $\Pi_i \Pi_j = 0$ for distinct outcomes $i, j \in \Gamma$.

Every POVM $\{M_1, \ldots, M_m\}$ has an associated measurement channel, which is the linear map $\Phi_M \in \mathrm{C}(\mathcal{X}, \mathcal{Y})$ defined as

$$\Phi_M(X) = \sum_{i=1}^{m} \langle M_i, X \rangle E_{i,i} \tag{8}$$

for every $X \in \mathrm{L}(\mathcal{X})$, where one defines $\mathcal{Y} = \mathbb{C}^m$. It is easy to check that Φ_M is completely positive and trace preserving. Measure-and-prepare channels are a generalization of the idea above and are considered often in this work.

Definition 1 (Measure-and-prepare channel). A measure-and-prepare (or entanglement-breaking) channel $\Phi \in \mathrm{C}(\mathcal{X}, \mathcal{Y})$ is a channel for which there is a choice of POVM $\{M_1, \ldots, M_m\} \subset \mathrm{Pos}(\mathcal{X})$ and density matrices $\rho_1, \ldots, \rho_m \in \mathrm{D}(\mathcal{Y})$ such that Φ may be expressed as

$$\Phi(X) = \sum_{i=1}^{m} \langle M_i, X \rangle \, \rho_i \tag{9}$$

for every $X \in \mathrm{L}(\mathcal{X})$. The channel Φ is said to be generated by the POVM $\{M_1, \ldots, M_m\}$ and the density matrices $\rho_1, \ldots, \rho_m$.

Note that there is not typically a unique choice of POVM or collection of density operators that generate a given measure-and-prepare channel. Recall that the condition that a channel $\Phi \in \mathrm{C}(\mathcal{X}, \mathcal{Y})$ be measure-and-prepare is equivalent to the condition that $J(\Phi) \in \mathrm{Sep}(\mathcal{X} : \mathcal{Y})$ (i.e., its Choi representation is a separable operator).

1.3 Compatibility of states, measurements, and channels

We now consider the problem of compatibility for quantum states.

Definition 2 (Quantum state marginal problem). Two states $\rho_1 \in \mathrm{D}(\mathcal{X} \otimes \mathcal{Y}_1)$ and $\rho_2 \in \mathrm{D}(\mathcal{X} \otimes \mathcal{Y}_2)$ are said to be compatible if there exists another state $\rho \in \mathrm{D}(\mathcal{X} \otimes \mathcal{Y}_1 \otimes \mathcal{Y}_2)$ satisfying

$$\mathrm{Tr}_{\mathcal{Y}_2}(\rho) = \rho_1 \quad \text{and} \quad \mathrm{Tr}_{\mathcal{Y}_1}(\rho) = \rho_2. \tag{10}$$

The state ρ is said to be the joint state for ρ_1 and ρ_2. Determining whether two overlapping states are compatible is known as the quantum state marginal problem.

Suppose that $\{M_1, \ldots, M_m\} \subset \mathrm{Pos}(\mathcal{X})$ and $\{N_1, \ldots, N_n\} \subset \mathrm{Pos}(\mathcal{X})$ are POVMs for some complex Euclidean space $\mathcal{X}$. One can ask whether it is possible to obtain the statistics of both measurements as course-grainings from a single measurement. If so, the measurements are said to be compatible.

Definition 3 (Measurement compatibility). POVMS $\{M_1, \ldots, M_m\} \subset \mathrm{Pos}(\mathcal{X})$ and $\{N_1, \ldots, N_n\} \subset \mathrm{Pos}(\mathcal{X})$ are said to be compatible if there exists another POVM of the form

$$\{P_{i,j} : 1 \leq i \leq m, 1 \leq j \leq n\} \subset \mathrm{Pos}(\mathcal{X}) \tag{11}$$

satisfying

$$M_i = \sum_{j=1}^{n} P_{i,j} \qquad \text{and} \qquad N_j = \sum_{i=1}^{m} P_{i,j} \tag{12}$$

for each choice of indices $i \in \{1, \ldots, m\}$ and $j \in \{1, \ldots, n\}$. A POVM P satisfying these conditions is said to be a compatibilizer (or compatibilizing measurement) for M and N.

Compatibility of POVMs has been investigated before (see [2] for a review). Analogous to the compatibility of POVMs, one may consider whether two channels may obtained as the marginals of another larger channel. This notion of compatibility is defined below.

Definition 4 (Channel compatibility). Two channels $\Phi_1 \in \mathrm{C}(\mathcal{X}, \mathcal{Y}_1)$ and $\Phi_2 \in \mathrm{C}(\mathcal{X}, \mathcal{Y}_2)$ are compatible if there exists a channel $\Phi \in \mathrm{C}(\mathcal{X}, \mathcal{Y}_1 \otimes \mathcal{Y}_2)$ satisfying

$$\Phi_1(X) = \mathrm{Tr}_{\mathcal{Y}_2}(\Phi(X)) \qquad \text{and} \qquad \Phi_2(X) = \mathrm{Tr}_{\mathcal{Y}_1}(\Phi(X)) \tag{13}$$

for every $X \in \mathrm{L}(\mathcal{X})$. The channel Φ is said to be a compatibilizer (or compatibilizing channel) for Φ_1 and Φ_2.

Compatibility of channels has also been investigated recently (see, e.g., [3, 4, 5, 6, 7]). Note that two compatible channels do not necessarily possess a unique compatibilizer. For a simple example of a pair of channels that do not possess a unique compatibilizer, consider the completely dephasing channel defined by the equation $\Omega(X) = \mathrm{Tr}(X)\mathbb{1}_{\mathcal{X}} / \dim(\mathcal{X})$. This channel is trivially self-compatible, but there are infinitely many choices compatibilizing channel for two copies of Ω. Indeed, any channel $\Phi \in \mathrm{T}(\mathcal{X}, \mathcal{X}_1 \otimes \mathcal{X}_2)$ having the form $\Phi(X) = \mathrm{Tr}(X)\rho$, where $\mathcal{X} = \mathcal{X}_1 = \mathcal{X}_2$ and $\rho \in \mathrm{D}(\mathcal{X}_1 \otimes \mathcal{X}_2)$ is a maximally entangled state, will compatibilize two copies of Ω. This follows from the fact that $\mathrm{Tr}_{\mathcal{X}_1}(\rho) = \mathrm{Tr}_{\mathcal{X}_2}(\rho) = \mathbb{1}_{\mathcal{X}} / \dim(\mathcal{X})$ holds for every choice of maximally entangled state $\rho \in \mathrm{D}(\mathcal{X}_1 \otimes \mathcal{X}_2)$. A channel $\Phi \in \mathrm{C}(\mathcal{X}, \mathcal{Y})$ is said to be self-compatible if two copies of Φ are compatible.

It is natural to generalize the notion of compatibility to more than two channels. In particular, for $k \geq 2$, a collection of channels $\Phi_1 \in \mathrm{C}(\mathcal{X}, \mathcal{Y}_1), \ldots, \Phi_k \in \mathrm{C}(\mathcal{X}, \mathcal{Y}_k)$ are said to be compatible if there exists a compatibilizing channel $\Phi \in \mathrm{C}(\mathcal{X}, \mathcal{Y}_1 \otimes \cdots \otimes \mathcal{Y}_k)$ such that

$$\mathrm{Tr}_{\mathcal{Y}_1 \otimes \cdots \otimes \mathcal{Y}_k \setminus \mathcal{Y}_a}(\Phi(X)) = \Phi_a(X) \tag{14}$$

holds for every $X \in \mathrm{L}(\mathcal{X})$ and each index $a \in \{1, \ldots, k\}$. A channel $\Phi \in \mathrm{C}(\mathcal{X}, \mathcal{Y})$ is said to be k-self-compatible if k copies of the channel Φ are compatible. It is known that a channel is measure and prepare if and only if it is k-self-compatible for every positive integer $k \in \mathbb{N}$.

Supplementary Note 2 - Equivalence of the quantum state and quantum channel versions of the marginal problem

It is straightforward to see that the marginal problem for quantum states generalizes the problem of determining compatibility for quantum channels. Indeed, two channels are compatible precisely when their corresponding normalized Choi representations are compatible as states [4, 8]. To see this, suppose a pair of channels $\Phi_1 \in \mathrm{C}(\mathcal{X}, \mathcal{Y}_1)$ and $\Phi_2 \in \mathrm{C}(\mathcal{X}, \mathcal{Y}_2)$ are compatible with some choice of compatibilizing channel $\Phi \in \mathrm{C}(\mathcal{X}, \mathcal{Y}_1 \otimes \mathcal{Y}_2)$. Consider now the condition that

$$\mathrm{Tr}_{\mathcal{Y}_2} \circ \Phi = \Phi_1. \tag{15}$$

The left- and right-hand side of this equality must be equal as maps, so their Choi representations must also coincide. Let ρ, ρ_1, and ρ_2 be the states defined by normalizing versions of the Choi representations of Φ, Φ_1, and Φ_2, respectively, i.e.,

$$\rho = \frac{1}{\dim(\mathcal{X})} J(\Phi), \qquad \rho_1 = \frac{1}{\dim(\mathcal{X}_1)} J(\Phi_1), \qquad \text{and} \qquad \rho_2 = \frac{1}{\dim(\mathcal{X})} J(\Phi_2). \tag{16}$$

Then the equality in Eq. (15) is equivalent to the condition tat

$$\mathrm{Tr}_{\mathcal{Y}_2}(\rho) = \rho_1. \tag{17}$$

Similarly, the condition that $\mathrm{Tr}_{\mathcal{Y}_1} \circ \Phi = \Phi_2$ is equivalent to the condition tat

$$\mathrm{Tr}_{\mathcal{Y}_1}(\rho) = \rho_2. \tag{18}$$

Hence the question of compatibility for a pair of quantum channels can be reduced to the corresponding marginal problem for the corresponding states defined as the normalized Choi representations.

It is also the case that the state marginal problem can be reduced to the channel compatibility problem. In particular, every quantum state marginal problem can be reformulated as the problem of determining compatibility of some pair of channels. This has already been established in [8] in the case when the marginals of the states have full rank. In this section, we prove this reduction holds even in the more general case when the marginals do not necessarily have full rank.

Let $\rho_1 \in \mathrm{D}(\mathcal{X} \otimes \mathcal{Y}_1)$ and $\rho_2 \in \mathrm{D}(\mathcal{X} \otimes \mathcal{Y}_2)$ be a pair of compatible quantum states. It must be the case that

$$\mathrm{Tr}_{\mathcal{Y}_1}(\rho_1) = \mathrm{Tr}_{\mathcal{Y}_1 \otimes \mathcal{Y}_2}(\rho) = \mathrm{Tr}_{\mathcal{Y}_2}(\rho_2), \tag{19}$$

where ρ is some choice of joint state. Therefore we may assume without loss of generality that

$$\mathrm{Tr}_{\mathcal{Y}_1}(\rho_1) = \mathrm{Tr}_{\mathcal{Y}_2}(\rho_2), \tag{20}$$

as otherwise the pair of states would be clearly not compatible. If it holds further that these marginals are both equal to $\mathrm{Tr}_{\mathcal{Y}_1}(\rho_1) = \mathrm{Tr}_{\mathcal{Y}_2}(\rho_2) = \mathbb{1}_{\mathcal{X}} / \dim(\mathcal{X})$ (i.e., the maximally mixed state), one may simply view ρ_1 and ρ_2 as the normalizations of the Choi representations for some channels $\Phi_1 \in \mathrm{C}(\mathcal{X}, \mathcal{Y}_1)$ and $\Phi_2 \in \mathrm{C}(\mathcal{X}, \mathcal{Y}_2)$. In this case, the marginal problem for ρ_1 and ρ_2 is trivially equivalent to the problem of determining the compatibility of the channels Φ_1 and Φ_2.

The more general case, when the marginal state $\mathrm{Tr}_{\mathcal{Y}_1}(\rho_1) = \mathrm{Tr}_{\mathcal{Y}_2}(\rho_2)$ is not proportional to the identity operator, is considered in Theorem 6. We first prove the following useful lemma, which will assist us in considering the case when the marginal does not have full rank.

Lemma 5. Let $A \in \mathrm{Pos}(\mathcal{X} \otimes \mathcal{Y})$ be a positive operator and let $\Pi := \Pi_{\mathrm{im}(\mathrm{Tr}_{\mathcal{Y}}(A))}$ be the projection operator onto $\mathrm{im}(\mathrm{Tr}_{\mathcal{Y}}(A)) \subseteq \mathcal{X}$. It holds that

$$A = (\Pi \otimes \mathbb{1}_{\mathcal{Y}})\, A\, (\Pi \otimes \mathbb{1}_{\mathcal{Y}}). \tag{21}$$

Proof. Note that

$$\langle A, (\mathbb{1}_{\mathcal{X}} - \Pi) \otimes \mathbb{1}_{\mathcal{Y}} \rangle = \langle \mathrm{Tr}_{\mathcal{Y}}(A), \mathbb{1}_{\mathcal{X}} - \Pi \rangle = 0. \tag{22}$$

As both A and $(\mathbb{1}_{\mathcal{X}} - \Pi) \otimes \mathbb{1}_{\mathcal{Y}}$ are positive operators, it follows that $A((\mathbb{1}_{\mathcal{X}} - \Pi) \otimes \mathbb{1}_{\mathcal{Y}}) = 0$ and $((\mathbb{1}_{\mathcal{X}} - \Pi) \otimes \mathbb{1}_{\mathcal{Y}})A = 0$. The desired result follows.

We are now ready to show that the marginal problem for any pair of states, $\rho_1 \in \mathrm{D}(\mathcal{X} \otimes \mathcal{Y}_1)$ and $\rho_2 \in \mathrm{D}(\mathcal{X} \otimes \mathcal{Y}_2)$, can be reduced to the compatibility problem for a particular pair of channels, $\Phi_1 \in \mathrm{C}(\mathcal{X}, \mathcal{Y}_1)$ and $\Phi_2 \in \mathrm{C}(\mathcal{X}, \mathcal{Y}_2)$ and.

Theorem 6. Let $\rho_1 \in \mathrm{D}(\mathcal{X} \otimes \mathcal{Y}_1)$ and $\rho_2 \in \mathrm{D}(\mathcal{X} \otimes \mathcal{Y}_2)$ be states and suppose there is a state $\sigma \in \mathrm{D}(\mathcal{X})$ satisfying

$$\sigma = \mathrm{Tr}_{\mathcal{Y}_1}(\rho_1) = \mathrm{Tr}_{\mathcal{Y}_2}(\rho_2). \tag{23}$$

Let $\Phi_1 \in \mathrm{T}(\mathcal{X}, \mathcal{Y}_1)$ and $\Phi_2 \in \mathrm{T}(\mathcal{X}, \mathcal{Y}_2)$ be the linear maps whose Choi representations may be expressed as

$$J(\Phi_1) = (\sigma^{-\frac{1}{2}} \otimes \mathbb{1}_{\mathcal{Y}_1})\rho_1(\sigma^{-\frac{1}{2}} \otimes \mathbb{1}_{\mathcal{Y}_1}) + \frac{1}{\dim(\mathcal{Y}_1)}(\mathbb{1}_{\mathcal{X}} - \Pi_{\mathrm{im}(\sigma)}) \otimes \mathbb{1}_{\mathcal{Y}_1} \tag{24}$$

and

$$J(\Phi_2) = (\sigma^{-\frac{1}{2}} \otimes \mathbb{1}_{\mathcal{Y}_2})\rho_2(\sigma^{-\frac{1}{2}} \otimes \mathbb{1}_{\mathcal{Y}_2}) + \frac{1}{\dim(\mathcal{Y}_2)}(\mathbb{1}_{\mathcal{X}} - \Pi_{\mathrm{im}(\sigma)}) \otimes \mathbb{1}_{\mathcal{Y}_2} \tag{25}$$

(where we interpret $\sigma^{-\frac{1}{2}}$ as the Moore–Penrose pseudoinverse of $\sigma^{\frac{1}{2}}$ if σ is not invertible). The maps Φ_1 and Φ_2 are channels. Moreover, the operators ρ_1 and ρ_2 are compatible as states if and only if Φ_1 and Φ_2 are compatible as channels.

Proof. Note that Φ_1 and Φ_2 are completely positive, as each of the terms in the sums on the right-hand sides of the equalities in Eq. (24) and Eq. (25) are positive semidefinite operators. These maps are also trace preserving, as

$$\mathrm{Tr}_{\mathcal{Y}_1}(J(\Phi_1)) = \sigma^{-\frac{1}{2}}\sigma\sigma^{-\frac{1}{2}} + \mathbb{1}_{\mathcal{X}} - \Pi_{\mathrm{im}(\sigma)} = \mathbb{1}_{\mathcal{X}} \tag{26}$$

(where we note that $\Pi_{\mathrm{im}(\sigma)} = \sigma^{-\frac{1}{2}}\sigma\sigma^{-\frac{1}{2}}$) and similarly $\mathrm{Tr}_{\mathcal{Y}_2}(J(\Phi_2)) = \mathbb{1}_{\mathcal{X}}$. We may conclude that the maps Φ_1 and Φ_2 are channels, as claimed.

Now suppose that ρ_1 and ρ_2 are compatible and let $\rho \in \mathrm{D}(\mathcal{X} \otimes \mathcal{Y}_1 \otimes \mathcal{Y}_2)$ be a choice of joint state for ρ_1 and ρ_2. Let $\Phi \in \mathrm{T}(\mathcal{X}, \mathcal{Y}_1 \otimes \mathcal{Y}_2)$ be the linear map whose Choi representation may be expressed as

$$\begin{aligned} J(\Phi) &= (\sigma^{-\frac{1}{2}} \otimes \mathbb{1}_{\mathcal{Y}_1} \otimes \mathbb{1}_{\mathcal{Y}_2})\rho(\sigma^{-\frac{1}{2}} \otimes \mathbb{1}_{\mathcal{Y}_1} \otimes \mathbb{1}_{\mathcal{Y}_2}) \\ &\quad + \frac{1}{\dim(\mathcal{Y}_1)\dim(\mathcal{Y}_2)}(\mathbb{1}_{\mathcal{X}} - \Pi_{\mathrm{im}(\sigma)}) \otimes \mathbb{1}_{\mathcal{Y}_1} \otimes \mathbb{1}_{\mathcal{Y}_2}. \end{aligned} \tag{27}$$

Note that Φ is completely positive, as its Choi representation is expressed in Eq. (27) as the sum of positive semidefinite operators. It is straightforward to verify that the map defined in this manner satisfies $\mathrm{Tr}_{\mathcal{Y}_2}(J(\Phi)) = J(\Phi_1)$ and $\mathrm{Tr}_{\mathcal{Y}_1}(J(\Phi)) = J(\Phi_2)$. Thus Φ is a compatibilizer for Φ_1 and Φ_2.

Suppose instead that Φ_1 and Φ_2 are compatible as channels and let $\Phi \in \mathrm{C}(\mathcal{X}, \mathcal{Y}_1 \otimes \mathcal{Y}_2)$ be a choice of compatibilizing channel. Define an operator $\rho \in \mathrm{L}(\mathcal{X} \otimes \mathcal{Y}_1 \otimes \mathcal{Y}_2)$ as

$$\rho = (\sigma^{\frac{1}{2}} \otimes \mathbb{1}_{\mathcal{Y}_1} \otimes \mathbb{1}_{\mathcal{Y}_2}) J(\Phi) (\sigma^{\frac{1}{2}} \otimes \mathbb{1}_{\mathcal{Y}_1} \otimes \mathbb{1}_{\mathcal{Y}_2}). \tag{28}$$

It is evident that ρ is positive semidefinite. Recalling the definition of Φ_1 from Eq. (24), one has that

$$\begin{aligned} \mathrm{Tr}_{\mathcal{Y}_2}(\rho) &= (\sigma^{\frac{1}{2}} \otimes \mathbb{1}_{\mathcal{Y}_1}) \mathrm{Tr}_{\mathcal{Y}_2}(J(\Phi)) (\sigma^{\frac{1}{2}} \otimes \mathbb{1}_{\mathcal{Y}_1}) \\ &= (\sigma^{\frac{1}{2}} \otimes \mathbb{1}_{\mathcal{Y}_1}) J(\Phi_1) (\sigma^{\frac{1}{2}} \otimes \mathbb{1}_{\mathcal{Y}_1}) \\ &= (\Pi_{\mathrm{im}(\sigma)} \otimes \mathbb{1}_{\mathcal{Y}_1}) \rho_1 (\Pi_{\mathrm{im}(\sigma)} \otimes \mathbb{1}_{\mathcal{Y}_1}) \\ &= \rho_1, \end{aligned} \tag{29}$$

where the equality in the third line follows from the facts that

$$\sigma^{\frac{1}{2}} \sigma^{-\frac{1}{2}} = \sigma^{-\frac{1}{2}} \sigma^{\frac{1}{2}} = \Pi_{\mathrm{im}(\sigma)} \qquad \text{and} \qquad \sigma^{\frac{1}{2}} (\mathbb{1}_{\mathcal{X}} - \Pi_{\mathrm{im}(\sigma)}) \sigma^{\frac{1}{2}} = 0, \tag{30}$$

and equality in the fourth line follows from Lemma 5. The proof that that $\mathrm{Tr}_{\mathcal{Y}_1}(\rho) = \rho_2$ is analogous. It follows that ρ is a joint state for ρ_1 and ρ_2, which completes the proof.

Supplementary Note 3 - Compatibility of measure-and-prepare channels

In this section, we consider some facts about the compatibility of measure-and-prepare channels (and also for the special case of measurement channels). We review some known conditions for compatibility of certain classes of measure-and-prepare channels. More importantly, we also consider the following question: If a pair of measure-and-prepare channels $\Phi_1 \in \mathrm{C}(\mathcal{X}, \mathcal{Y}_1)$ and $\Phi_2 \in \mathrm{C}(\mathcal{X}, \mathcal{Y}_2)$ are compatible, do they they necessarily possess a measure-and-prepare compatibilizer? We show that this is not the case by providing a counterexample.

3.1 Compatibility with measurement channels

Here we state a necessary and sufficient condition for a channel to be compatible with a fixed choice of measurement channel. Recall that a measurement channel is a special instance of a measure-and-prepare channel, where the measurement outcomes are recorded in the computation basis. The equivalence in the following proposition was shown in the more general context of generalized probability theories in [9, Lemma 1].

Proposition 7 ([9]). Let $\{M_1, \ldots, M_m\} \subset \mathrm{Pos}(\mathcal{X})$ be a POVM and let Φ_M be the measurement channel Φ_M generated by M,

$$\Phi_M(X) = \sum_{i=1}^{m} \langle M_i, X \rangle E_{i,i}. \tag{31}$$

Let $\Phi \in \mathrm{C}(\mathcal{X}, \mathcal{Y})$ be a channel. The following are equivalent.

1. The channels Φ and Φ_M are compatible.
2. There exist completely positive maps $\Phi_1, \dots, \Phi_m \in \mathrm{T}(\mathcal{X}, \mathcal{Y})$ satisfying $\Phi = \sum_{i=1}^m \Phi_i$ such that, for each $i \in \{1, \dots, m\}$, one has $\mathrm{Tr}(\Phi_i(X)) = \langle M_i, X \rangle$ for every $X \in \mathrm{L}(\mathcal{X})$.

We remark that the condition that $\mathrm{Tr}(\Phi_i(X)) = \langle M_i, X \rangle$ holds for every $X \in \mathrm{L}(\mathcal{X})$ is equivalent to the condition that $\mathrm{Tr}_{\mathcal{Y}}(J(\Phi_i)) = M_i^T$. Indeed, one has that

$$\mathrm{Tr}(\Phi_i(X)) = \langle J(\Phi_i), X^T \otimes \mathbb{1}_{\mathcal{Y}} \rangle = \langle \mathrm{Tr}_{\mathcal{Y}}(J(\Phi_i))^T, X \rangle. \tag{32}$$

for every $X \in \mathrm{L}(\mathcal{X})$, and the desired equivalence follows.

3.2 Compatibility of measure-and-prepare channels

Here we review some known results regarding the compatibility of measure-and-prepare channels, which are stated rigorously in Proposition 8. The first statement is that two measure-and-prepare channels are compatible as channels whenever their underlying POVMs are compatible as measurements. A stronger result holds if the collections of preparation states defining the measure-and-prepare channels are assumed to be distinguishable. Allow us to take a moment to recall what this means. A collection of density operators $\{\rho_1, \dots, \rho_m\} \subset \mathrm{D}(\mathcal{Y})$ is said to be (perfectly) distinguishable if it holds that $\rho_i \rho_j = 0$ for every pair of indices $i, j \in \{1, \dots, n\}$ satisfying $i \neq j$. In particular, this means that there exists a POVM that can perfectly distinguish between these states and, moreover, that such a POVM can be given by the projection operators, i.e, it is given as $\{\Pi_{\mathrm{im}(\rho_1)}, \dots, \Pi_{\mathrm{im}(\rho_m)}\}$, where the projections must be pairwise orthogonal.

Proposition 8. Let $\Phi_1 \in \mathrm{C}(\mathcal{X}, \mathcal{Y}_1)$ and $\Phi_2 \in \mathrm{C}(\mathcal{X}, \mathcal{Y}_2)$ be measure-and-prepare channels having the form

$$\Phi_1(X) = \sum_{i=1}^m \langle M_i, X \rangle \rho_i \qquad \text{and} \qquad \Phi_1(X) = \sum_{j=1}^n \langle N_j, X \rangle \sigma_j \tag{33}$$

for some choice of POVMs $\{M_1, \dots, M_m\} \subset \mathrm{Pos}(\mathcal{X})$ and $\{N_1, \dots, N_n\} \subset \mathrm{Pos}(\mathcal{X})$, and some collections of density matrices $\rho_1, \dots, \rho_m \in \mathrm{D}(\mathcal{Y}_1)$ and $\sigma_1, \dots, \sigma_n \in \mathrm{D}(\mathcal{Y}_2)$. The following statements hold:

(1) If M and N are compatible as POVMS then Φ_1 and Φ_2 are compatible as channels.

(2) If the collections of density matrices $\rho_1, \dots, \rho_m$ and $\sigma_1, \dots, \sigma_n$ are each distinguishable then the channels Φ_1 and Φ_2 are compatible if and only if M and N are compatible as POVMs.

Proof. To prove statement (1), suppose that M and N are compatible and let

$$\{P_{i,j} : 1 \leq i \leq m, 1 \leq j \leq n\} \subset \mathrm{Pos}(\mathcal{X}) \tag{34}$$

be a choice of POVM that compatibilizes M and N. Define a channel $\Phi \in \mathrm{C}(\mathcal{X}, \mathcal{Y}_1 \otimes \mathcal{Y}_2)$ as

$$\Phi(X) = \sum_{i=1}^m \sum_{j=1}^n \langle P_{i,j}, X \rangle \rho_i \otimes \sigma_j \tag{35}$$

for every $X \in \mathrm{L}(\mathcal{X})$. Taking partial traces, it may be verified that Φ compatibilizes Φ_1 and Φ_2.

One direction of statement (2) follows from statement (1). To prove the reverse implication, assume that the channels Φ_1 and Φ_2 are compatible and let $\Phi \in \mathrm{C}(\mathcal{X}, \mathcal{Y}_1 \otimes \mathcal{Y}_2)$ be a compatibilizing channel. For each choice of indices $i \in \{1, \ldots, m\}$ and $j \in \{1, \ldots, n\}$, one may define the operator

$$P_{i,j} = \Phi^*(\Pi_{\mathrm{im}(\rho_i)} \otimes \Pi_{\mathrm{im}(\sigma_j)}) \in \mathrm{Pos}(\mathcal{X}). \tag{36}$$

We may assume without loss of generality that $\sum_{i=1}^{m} \Pi_{\mathrm{im}(\rho_i)} = \mathbb{1}_{\mathcal{Y}_1}$. Indeed, the projection operators $\Pi_{\mathrm{im}(\rho_1)}, \ldots, \Pi_{\mathrm{im}(\rho_m)}$ must be pairwise orthogonal, as it has been assumed that the density matrices $\rho_1, \ldots, \rho_m$ are distinguishable, and if these projections did not sum to the identity, the operator $\Pi = \mathbb{1}_{\mathcal{X}} - \sum_{i=1}^{m} \Pi_{\mathrm{im}(\rho_i)}$ would be a nontrivial projection operator. One could then define the operator $M_{m+1} = 0$ and the state $\rho_{m+1} = \Pi / \operatorname{Tr}(\Pi)$ such that the POVM $\{M_1, \ldots, M_{m+1}\}$ and collection of density matrices $\rho_1, \ldots, \rho_{m+1}$ satisfy the desired property and generate the same measurement channel, which justifies our assumption. Similarly, it may be assumed without loss of generality that $\sum_{j=1}^{n} \Pi_{\mathrm{im}(\sigma_j)} = \mathbb{1}_{\mathcal{Y}_2}$.

For each index $i \in \{1, \ldots, m\}$, one has that

$$\sum_{j=1}^{n} \langle P_{i,j}, X \rangle = \langle \Pi_{\mathrm{im}(\rho_i)} \otimes \mathbb{1}_{\mathcal{Y}_2}, \Phi(X) \rangle = \langle \Pi_{\mathrm{im}(\rho_i)}, \Phi_1(X) \rangle = \langle M_i, X \rangle \tag{37}$$

every $X \in \mathrm{L}(\mathcal{X})$, and thus $\sum_{j=1}^{n} P_{i,j} = M_i$. Similarly, for each index $j \in \{1, \ldots, n\}$,

$$\sum_{i=1}^{m} \langle P_{i,j}, X \rangle = \langle \mathbb{1}_{\mathcal{Y}_1} \otimes \Pi_{\mathrm{im}(\sigma_j)}), \Phi(X) \rangle = \langle \Pi_{\mathrm{im}(\sigma_j)}, \Phi_2(X) \rangle = \langle N_j, X \rangle \tag{38}$$

holds for every $X \in \mathrm{L}(\mathcal{X})$, and thus $\sum_{i=1}^{m} P_{i,j} = N_j$. From these two conditions, it can be verified that P is a compatibilizing POVM for M and N, so the POVMs are compatible.

3.3 Compatibilizers of measure-and-prepare channels

Every measure-and-prepare channel $\Phi \in \mathrm{C}(\mathcal{X}, \mathcal{Y})$ is necessarily self-compatible and, moreover, there is a measure-and-prepare channel $\Psi \in \mathrm{C}(\mathcal{X}, \mathcal{Y} \otimes \mathcal{Y})$ that compatibilizes two copies of Φ. Indeed, suppose $\{M_1, \ldots, M_m\} \subset \mathrm{Pos}(\mathcal{X})$ is a POVM and $\rho_1, \ldots, \rho_m \in \mathrm{D}(\mathcal{Y})$ are density matrices such that Φ may be expressed as

$$\Phi(X) = \sum_{i=1}^{m} \langle M_i, X \rangle \, \rho_i. \tag{39}$$

One may define a measure-and-prepare compatibilizer $\Psi \in \mathrm{C}(\mathcal{X}, \mathcal{Y} \otimes \mathcal{Y})$ as

$$\Psi(X) = \sum_{i=1}^{m} \langle M_i, X \rangle \, \rho_i \otimes \rho_i. \tag{40}$$

We now consider the question of whether two compatible measure-and-prepare channels necessarily possess a measure-and-prepare compatibilizer. It turns out that the answer is no, which we demonstrate with the following example.

Example 9. Let $\mathcal{X} = \mathcal{Y}_1 = \mathcal{Y}_2 = \mathbb{C}^2$ and let $\Phi_1 \in \mathrm{C}(\mathcal{X}, \mathcal{Y}_1)$ and $\Phi_2 \in \mathrm{C}(\mathcal{X}, \mathcal{Y}_2)$ be the channels whose Choi representations $J(\Phi_1) \in \mathrm{L}(\mathcal{X} \otimes \mathcal{Y}_1)$ and $J(\Phi_2) \in \mathrm{L}(\mathcal{X} \otimes \mathcal{Y}_2)$ are given by the 4×4

matrices

$$J(\Phi_1) = \begin{pmatrix} \frac{3}{4} & \cdot & \cdot & \frac{1}{4} \\ \cdot & \frac{1}{4} & \cdot & \cdot \\ \cdot & \cdot & \frac{1}{4} & \cdot \\ \frac{1}{4} & \cdot & \cdot & \frac{3}{4} \end{pmatrix} \quad \text{and} \quad J(\Phi_2) = \begin{pmatrix} \frac{5}{8} & \cdot & \cdot & \frac{3}{8} \\ \cdot & \frac{3}{8} & \frac{1}{8} & \cdot \\ \cdot & \frac{1}{8} & \frac{3}{8} & \cdot \\ \frac{3}{8} & \cdot & \cdot & \frac{5}{8} \end{pmatrix}, \tag{41}$$

where $\cdot$ indicates an entry that is 0. One may observe that both $J(\Phi_1)$ and $J(\Phi_2)$ as well as their partial transposes (where $Z^{T_{\mathcal{X}}}$ denotes the partial transpose of an operator $Z \in \mathrm{L}(\mathcal{X} \otimes \mathcal{Y})$),

$$J(\Phi_1)^{T_{\mathcal{X}}} = \begin{pmatrix} \frac{3}{4} & \cdot & \cdot & \cdot \\ \cdot & \frac{1}{4} & \frac{1}{4} & \cdot \\ \cdot & \frac{1}{4} & \frac{1}{4} & \cdot \\ \cdot & \cdot & \cdot & \frac{3}{4} \end{pmatrix} \quad \text{and} \quad J(\Phi_2)^{T_{\mathcal{X}}} = \begin{pmatrix} \frac{5}{8} & \cdot & \cdot & \frac{1}{8} \\ \cdot & \frac{3}{8} & \frac{3}{8} & \cdot \\ \cdot & \frac{3}{8} & \frac{3}{8} & \cdot \\ \frac{1}{8} & \cdot & \cdot & \frac{5}{8} \end{pmatrix} \tag{42}$$

are positive semidefinite matrices. The operators $J(\Phi_1)$ and $J(\Phi_2)$ are therefore positive under partial transpose (PPT). It follows that Φ_1 and Φ_2 are necessarily measure-and-prepare channels as their Choi representations are separable as operators. Recall that a channel is measure-and-prepare channel if and only if its Choi representation is a separable operator. Moreover, for bipartite operators where each local dimension is equal to 2, an operator is separable if and only if it is PPT [10]. Consider now the map $\Phi \in \mathrm{T}(\mathcal{X}, \mathcal{Y}_1 \otimes \mathcal{Y}_2)$ whose Choi representation $J(\Phi) \in \mathrm{L}(\mathcal{X} \otimes \mathcal{Y}_1 \otimes \mathcal{Y}_2)$ is given by the 8×8 matrix

$$J(\Phi) = \begin{pmatrix} \frac{1}{2} & \cdot & \cdot & \cdot & \cdot & \frac{3}{16} & \frac{1}{8} & \cdot \\ \cdot & \frac{1}{4} & \cdot & \cdot & \frac{1}{16} & \cdot & \cdot & \frac{1}{8} \\ \cdot & \cdot & \frac{1}{8} & \cdot & \cdot & \cdot & \cdot & \frac{3}{16} \\ \cdot & \cdot & \cdot & \frac{1}{8} & \cdot & \cdot & \frac{1}{16} & \cdot \\ \cdot & \frac{1}{16} & \cdot & \cdot & \frac{1}{8} & \cdot & \cdot & \cdot \\ \frac{3}{16} & \cdot & \cdot & \cdot & \cdot & \frac{1}{8} & \cdot & \cdot \\ \frac{1}{8} & \cdot & \cdot & \frac{1}{16} & \cdot & \cdot & \frac{1}{4} & \cdot \\ \cdot & \frac{1}{8} & \frac{3}{16} & \cdot & \cdot & \cdot & \cdot & \frac{1}{2} \end{pmatrix}. \tag{43}$$

It is easy to verify that this map satisfies $\mathrm{Tr}_{\mathcal{Y}_2}(J(\Phi)) = J(\Phi_1)$ and $\mathrm{Tr}_{\mathcal{Y}_1}(J(\Phi)) = J(\Phi_2)$. Moreover, it may be verified that $J(\Phi)$ is positive definite, as the eigenvalues of $J(\Phi)$ are the four possible values of

$$\frac{4 \pm \sqrt{3} + \sqrt{10 \pm 4\sqrt{3}}}{16}, \tag{44}$$

each having multiplicity 2. Therefore Φ is a channel that compatibilizes Φ_1 and Φ_2, and thus the measure-and-prepare channels Φ_1 and Φ_2 are compatible.

It remains to show that there does not exist a measure-and-prepare compatibilizer for the channels Φ_1 and Φ_2. Toward a contradiction, suppose there exists some choice of measure-and-prepare channel $\Psi \in \mathrm{C}(\mathcal{X}, \mathcal{Y}_1 \otimes \mathcal{Y}_2)$ that compatibilizes the channels Φ_1 and Φ_2. Using the fact that every separable operator must be PPT, this channel must satisfy

$$\mathrm{Tr}_{\mathcal{Y}_2}(J(\Psi)) = J(\Phi_1), \qquad \mathrm{Tr}_{\mathcal{Y}_1}(J(\Psi)) = J(\Phi_2), \qquad \text{and} \qquad J(\Psi)^{T_{\mathcal{X}}} \geq 0. \tag{45}$$

For any choice of Hermitian operators $Z_1 \in \mathrm{L}(\mathcal{X} \otimes \mathcal{Y}_1)$ and $Z_2 \in \mathrm{L}(\mathcal{X} \otimes \mathcal{Y}_2)$ satisfying

$$\mathrm{Tr}^*_{\mathcal{Y}_2}(Z_1) + \mathrm{Tr}^*_{\mathcal{Y}_1}(Z_2) \geq 0, \tag{46}$$

it must be the case that

$$\begin{aligned}\langle J(\Phi_1)^{T_\mathcal{X}}, Z_1\rangle + \langle J(\Phi_2)^{T_\mathcal{X}}, Z_2\rangle &= \langle \mathrm{Tr}_{\mathcal{Y}_2}(J(\Psi)^{T_\mathcal{X}}), Z_1\rangle + \langle \mathrm{Tr}_{\mathcal{Y}_1}(J(\Psi)^{T_\mathcal{X}}), Z_2\rangle \\ &= \langle J(\Psi)^{T_\mathcal{X}}, \mathrm{Tr}^*_{\mathcal{Y}_2}(Z_1) + \mathrm{Tr}^*_{\mathcal{Y}_1}(Z_2)\rangle \\ &\geq 0,\end{aligned} \tag{47}$$

where the inequality follows from the fact that the expression in the second line is an inner product of two operators that are positive semidefinite by assumption, and Ψ is any channel that satisfies the conditions in Eq. (45). (Here we make use of the adjoint maps $\mathrm{Tr}^*_{\mathcal{Y}_1}$ and $\mathrm{Tr}^*_{\mathcal{Y}_2}$ of the corresponding partial traces. To preserve the correct ordering of the spaces in the tensor product, these are defined here as the maps that satisfy

$$\mathrm{Tr}^*_{\mathcal{Y}_1}(X \otimes Y_2) = X \otimes \mathbb{1}_{\mathcal{Y}_1} \otimes Y_2 \qquad \text{and} \qquad \mathrm{Tr}^*_{\mathcal{Y}_2}(X \otimes Y_1) = X \otimes Y_1 \otimes \mathbb{1}_{\mathcal{Y}_2} \tag{48}$$

for every $X \in \mathrm{L}(\mathcal{X})$, $Y_1 \in \mathrm{L}(\mathcal{Y}_1)$, and $Y_2 \in \mathrm{L}(\mathcal{Y}_2)$.)

To prove that there does not exist a measure-and-prepare compatibilizer, it therefore suffices to find a choice of operators $Z_1 \in \mathrm{L}(\mathcal{X} \otimes \mathcal{Y}_1)$ and $Z_2 \in \mathrm{L}(\mathcal{X} \otimes \mathcal{Y}_2)$ satisfying

$$\mathrm{Tr}^*_{\mathcal{Y}_2}(Z_1) + \mathrm{Tr}^*_{\mathcal{Y}_1}(Z_2) \geq 0 \qquad \text{and} \qquad \langle J(\Phi_1)^{T_\mathcal{X}}, Z_1\rangle + \langle J(\Phi_2)^{T_\mathcal{X}}, Z_2\rangle < 0. \tag{49}$$

Toward this goal, let Z_1 and Z_2 be the operators defined by the matrices

$$Z_1 = \begin{pmatrix} -2 & \cdot & \cdot & 2 \\ \cdot & 48 & -38 & \cdot \\ \cdot & -38 & 48 & \cdot \\ 2 & \cdot & \cdot & -2 \end{pmatrix} \qquad \text{and} \qquad Z_2 = \begin{pmatrix} 3 & \cdot & \cdot & -4 \\ \cdot & 40 & -47 & \cdot \\ \cdot & -47 & 40 & \cdot \\ -4 & \cdot & \cdot & 3 \end{pmatrix}. \tag{50}$$

For this choice of operators, one has that

$$\langle J(\Phi_1)^{T_\mathcal{X}}, Z_1\rangle + \langle J(\Phi_2)^{T_\mathcal{X}}, Z_2\rangle = -\frac{1}{2} < 0. \tag{51}$$

However, the matrix

$$\mathrm{Tr}^*_{\mathcal{Y}_2}(Z_1) + \mathrm{Tr}^*_{\mathcal{Y}_1}(Z_2) = \begin{pmatrix} 1 & \cdot & \cdot & \cdot & \cdot & -4 & 2 & \cdot \\ \cdot & 38 & \cdot & \cdot & -47 & \cdot & \cdot & 2 \\ \cdot & \cdot & 51 & \cdot & -38 & \cdot & \cdot & -4 \\ \cdot & \cdot & \cdot & 88 & \cdot & -38 & -47 & \cdot \\ \cdot & -47 & -38 & \cdot & 88 & \cdot & \cdot & \cdot \\ -4 & \cdot & \cdot & -38 & \cdot & 51 & \cdot & \cdot \\ 2 & \cdot & \cdot & -47 & \cdot & \cdot & 38 & \cdot \\ \cdot & 2 & -4 & \cdot & \cdot & \cdot & \cdot & 1 \end{pmatrix} \tag{52}$$

may be verified to be positive semidefinite. This contradicts the assumption that there exists a measure-and-prepare compatibilizer for Φ_1 and Φ_2, and thus no such compatiblizer exists.

Remark 10. The matrices above where found numerically using the semidefinite programming formulations discussed later in this work. However, the proof above does not rely on semidefinite programming and can be presented without this formalism.

Supplementary Note 4 - The Jordan product of channels

For any pair of operators $A, B \in \mathrm{L}(\mathcal{X})$, their Jordan product is defined as

$$A \odot B := \frac{1}{2}(AB + BA). \tag{53}$$

The Jordan product provides a useful sufficient condition for verifying that two POVMs are compatible. Let $\{M_1, \ldots, M_m\} \subset \mathrm{Pos}(\mathcal{X})$ and $\{N_1, \ldots, N_n\} \subset \mathrm{Pos}(\mathcal{X})$ be two POVMs. If it holds that

$$M_i \odot N_j \geq 0 \tag{54}$$

for each pair of indices i and j, then M and N are compatible. Indeed, one may define a choice of compatibilizing POVM as $P_{i,j} = M_i \odot N_j$ [11]. If at least one of the POVMs M or N is also a projection-valued measure (PVM), then the condition in Eq. (54) is also a necessary condition for these POVMs to be compatible [12].

In this section, we generalize the notion of Jordan products for operators to linear maps using the Choi representation. We also investigate properties of Jordan products of quantum channels.

4.1 Definition and properties

Here we define the notion of Jordan products of linear maps and discuss a few of its properties.

Definition 11 (Jordan product of linear maps). Let $\Phi_1 \in \mathrm{T}(\mathcal{X}, \mathcal{Y}_1)$ and $\Phi_2 \in \mathrm{T}(\mathcal{X}, \mathcal{Y}_2)$ be linear maps. The Jordan product of Φ_1 and Φ_2 is the linear map

$$\Phi_1 \odot \Phi_2 \in \mathrm{T}(\mathcal{X}, \mathcal{Y}_1 \otimes \mathcal{Y}_2) \tag{55}$$

whose Choi representation $J(\Phi_1 \odot \Phi_2) \in \mathrm{L}(\mathcal{X} \otimes \mathcal{Y}_1 \otimes \mathcal{Y}_2)$ is the operator

$$J(\Phi_1 \odot \Phi_2) := \sum_{i,j,k,\ell=1}^{\dim(\mathcal{X})} (E_{i,j} \odot E_{k,\ell}) \otimes \Phi_1(E_{i,j}) \otimes \Phi_2(E_{k,\ell}). \tag{56}$$

Many useful properties of the Jordan product for linear maps follow immediately from the definition, some of which are outlined as follows. The binary operation $\odot$ is bilinear—that is, for all choices of linear maps $\Phi_1, \Phi_1' \in \mathrm{T}(\mathcal{X}, \mathcal{Y}_1)$ and $\Phi_2, \Phi_2' \in \mathrm{T}(\mathcal{X}, \mathcal{Y}_2)$ and scalars $\alpha, \beta \in \mathbb{C}$, one has that

$$(\alpha\Phi_1 + \beta\Phi_1') \odot \Phi_2 = \alpha\, \Phi_1 \odot \Phi_2 + \beta\, \Phi_1' \odot \Phi_2 \tag{57}$$

and

$$\Phi_1 \odot (\alpha\Phi_2 + \beta\Phi_2') = \alpha\, \Phi_1 \odot \Phi_2 + \beta\, \Phi_1 \odot \Phi_2'. \tag{58}$$

For any further choices of linear maps $\Psi_1 \in \mathrm{T}(\mathcal{Y}_1, \mathcal{Y}_1')$ and $\Psi_2 \in \mathrm{T}(\mathcal{Y}_2, \mathcal{Y}_2')$, one has that

$$(\Psi_1 \otimes \Psi_2) \circ (\Phi_1 \odot \Phi_2) = (\Psi_1 \circ \Phi_1) \odot (\Psi_2 \circ \Phi_2). \tag{59}$$

It is also evident that

$$(\Phi_2 \odot \Phi_1)(X) = \mathrm{Swap}_{\mathcal{Y}_1, \mathcal{Y}_2}\big((\Phi_1 \odot \Phi_2)(X)\big) \tag{60}$$

holds for every $X \in \mathrm{L}(\mathcal{X})$, where $\mathrm{Swap}_{\mathcal{Y}_1,\mathcal{Y}_2} \in \mathrm{T}(\mathcal{Y}_1 \otimes \mathcal{Y}_2, \mathcal{Y}_2 \otimes \mathcal{Y}_1)$ is the linear map defined by the equation

$$\mathrm{Swap}_{\mathcal{Y}_1,\mathcal{Y}_2}(Y_1 \otimes Y_2) = Y_2 \otimes Y_1 \tag{61}$$

for every $Y_1 \in \mathrm{L}(\mathcal{Y}_1)$ and $Y_2 \in \mathrm{L}(\mathcal{Y}_2)$.

It is useful to note a few alternative ways of representing this Jordan product. In particular, the Choi representation of the Jordan product of $\Phi_1 \in \mathrm{T}(\mathcal{X}, \mathcal{Y}_1)$ and $\Phi_2 \in \mathrm{T}(\mathcal{X}, \mathcal{Y}_2)$ can be expressed as

$$J(\Phi_1 \odot \Phi_2) = \frac{1}{2} \sum_{i,j=1}^{\dim(\mathcal{X})} E_{i,j} \otimes \left(\sum_{k=1}^{\dim(\mathcal{X})} \Phi_1(E_{i,k}) \otimes \Phi_2(E_{k,j}) + \Phi_1(E_{k,j}) \otimes \Phi_2(E_{i,k}) \right). \tag{62}$$

It is then straightforward to check that

$$(\Phi_1 \odot \Phi_2)(X) = \frac{1}{2}(\Phi_1 \otimes \Phi_2)\big(W(X \otimes \mathbb{1}_{\mathcal{X}} + \mathbb{1}_{\mathcal{X}} \otimes X)\big) \tag{63}$$

for every $X \in \mathrm{L}(\mathcal{X})$, where $W \in \mathrm{U}(\mathcal{X} \otimes \mathcal{X})$ is the unitary operator defined by the equation

$$W(x \otimes y) = y \otimes x \quad \text{for every} \quad x, y \in \mathcal{X}. \tag{64}$$

Indeed, making use of the expression in Eq. (62), one has

$$\begin{aligned}
(\Phi_1 \odot \Phi_2)(X) &= \mathrm{Tr}_{\mathcal{X}}\big(J(\Phi_1 \odot \Phi_2)(X^T \otimes \mathbb{1}_{\mathcal{Y}_1 \otimes \mathcal{Y}_2})\big) \\
&= \frac{1}{2}(\Phi_1 \otimes \Phi_2) \left(\sum_{i,j,k=1}^{\dim(\mathcal{X})} \langle E_{i,j}, X \rangle (E_{i,k} \otimes E_{k,j} + E_{k,j} \otimes E_{i,k}) \right) \\
&= \frac{1}{2}(\Phi_1 \otimes \Phi_2) \left(\sum_{i,j,k=1}^{\dim(\mathcal{X})} \langle E_{i,j}, X \rangle W(E_{k,k} \otimes E_{i,j} + E_{i,j} \otimes E_{k,k}) \right) \\
&= \frac{1}{2}(\Phi_1 \otimes \Phi_2)(W(\mathbb{1}_{\mathcal{X}} \otimes X + X \otimes \mathbb{1}_{\mathcal{X}}))
\end{aligned} \tag{65}$$

where we have used the identity $X = \sum_{i,j=1}^{\dim(\mathcal{X})} \langle E_{i,j}, X \rangle E_{i,j}$.

Other interesting properties of Jordan products arise when considering the Jordan product of channels. In particular, the Jordan product of trace-preserving maps is also trace preserving, as the following proposition shows.

Proposition 12. Suppose that $\Phi_1 \in \mathrm{T}(\mathcal{X}, \mathcal{Y}_1)$ and $\Phi_2 \in \mathrm{T}(\mathcal{X}, \mathcal{Y}_2)$ are trace-preserving maps. One has that

$$\mathrm{Tr}_{\mathcal{Y}_2}(J(\Phi_1 \odot \Phi_2)) = J(\Phi_1) \quad \text{and} \quad \mathrm{Tr}_{\mathcal{Y}_1}(J(\Phi_1 \odot \Phi_2)) = J(\Phi_2). \tag{66}$$

Moreover, $\Phi_1 \odot \Phi_2$ is trace preserving.

Proof. Using Eq. (62), we can write

$$\begin{aligned}
\mathrm{Tr}_{\mathcal{Y}_2}(J(\Phi_1 \odot \Phi_2)) &= \frac{1}{2} \sum_{i,j=1}^{\dim(\mathcal{X})} E_{i,j} \otimes \left(\sum_{k=1}^{\dim(\mathcal{X})} \mathrm{Tr}(\Phi_2(E_{k,j}))\Phi_1(E_{i,k}) + \mathrm{Tr}(\Phi_2(E_{i,k}))\Phi_1(E_{k,j}) \right) \\
&= \frac{1}{2} \sum_{i,j=1}^{\dim(\mathcal{X})} E_{i,j} \otimes \big(\Phi_1(E_{i,j}) + \Phi_1(E_{i,j})\big) \\
&= J(\Phi_1),
\end{aligned} \tag{67}$$

where we made use of the fact that Φ_2 is trace preserving. The proof of the other equality in Eq. (66) is analogous. Finally, note that

$$\mathrm{Tr}_{\mathcal{Y}_1 \otimes \mathcal{Y}_2}(J(\Phi_1 \odot \Phi_2)) = \mathrm{Tr}_{\mathcal{Y}_1}(J(\Phi_1)) = \mathbb{1}_{\mathcal{X}} \tag{68}$$

and thus $\Phi_1 \odot \Phi_2$ is trace preserving.

An immediate consequence of Proposition 12 is that the Jordan product provides us with a useful criteria for determining if two channels are compatible. In particular, given two channels $\Phi_1 \in \mathrm{C}(\mathcal{X}, \mathcal{Y}_1)$ and $\Phi_2 \in \mathrm{C}(\mathcal{X}, \mathcal{Y}_2)$, one straightforward way to check if Φ_1 and Φ_2 are compatible is to check if the Jordan product map $\Phi_1 \odot \Phi_2$ is completely positive.

Corollary 13. Suppose that $\Phi_1 \in \mathrm{C}(\mathcal{X}, \mathcal{Y}_1)$ and $\Phi_2 \in \mathrm{C}(\mathcal{X}, \mathcal{Y}_2)$ are such that $\Phi_1 \odot \Phi_2$ is completely positive. Then $\Phi_1 \odot \Phi_2$ compatibilizes Φ_1 and Φ_2.

As this is only a sufficient condition, other methods must be used to determine the compatibility of the channels whose Jordan product is not completely positive.

If the maps Φ_1 and Φ_2 are both measure-and-prepare channels, the Jordan product map $\Phi_1 \odot \Phi_2$ can be obtained simply by taking the Jordan products of the elements of the POVMs that generate Φ_1 and Φ_2, discussed in the proposition below. It is in this sense that the sufficient condition for the compatibility of measurements via the Jordan product of the POVM elements is a generalization of the similar sufficient condition via the Jordan product for channels.

Proposition 14. Let $\Phi_1 \in \mathrm{C}(\mathcal{X}, \mathcal{Y}_1)$ and $\Phi_2 \in \mathrm{C}(\mathcal{X}, \mathcal{Y}_2)$ be measure-and-prepare channels that are generated by the POVMs $\{M_1, \ldots, M_m\} \subset \mathrm{Pos}(\mathcal{X})$ and $\{N_1, \ldots, N_n\} \subset \mathrm{Pos}(\mathcal{X})$, and the collections of density matrices $\rho_1, \ldots, \rho_m \in \mathrm{D}(\mathcal{Y}_1)$ and $\sigma_1, \ldots, \sigma_n \in \mathrm{D}(\mathcal{Y}_2)$, respectively. It holds that

$$J(\Phi_1 \odot \Phi_2) = \sum_{i=1}^{m} \sum_{j=1}^{n} \left(M_i \odot N_j\right)^T \otimes \rho_i \otimes \sigma_j. \tag{69}$$

Moreover, if $M_i \odot N_j \geq 0$ for each index i and j, then the map $\Phi_1 \odot \Phi_2$ is completely positive (and thus compatible).

Proof. This follows by direct calculation from the definition of the Jordan product of maps.

There are a few further properties of Jordan products of channels that follow directly from the definition that we mention here. Given channels $\Phi_1, \Phi_1' \in \mathrm{C}(\mathcal{X}, \mathcal{Y}_1)$, a channel $\Phi_2 \in \mathrm{C}(\mathcal{X}, \mathcal{Y}_2)$, and a scalar $\lambda \in [0, 1]$, if it holds that $J(\Phi_1 \odot \Phi_2) \geq 0$ and $J(\Phi_1' \odot \Phi_2) \geq 0$ then

$$J((\lambda \Phi_1 + (1 - \lambda)\Phi_1') \odot \Phi_2) \geq 0. \tag{70}$$

Furthermore, for any other choice of channel $\Psi \in \mathrm{C}(\mathcal{Y}_1, \mathcal{Y}_1')$, if it holds that $J(\Phi_1 \odot \Phi_2) \geq 0$ then $J((\Psi \circ \Phi_1) \odot \Phi_2) \geq 0$ as well.

4.2 Conditions for the compatibility from the Jordan product

Recall that a projection-valued measure (PVM) is a collection $\Pi = \{\Pi_1, \ldots, \Pi_m\}$ of projection operators that satisfy $\sum_{i=1}^{m} \Pi_i = \mathbb{1}_{\mathcal{X}}$. Let $\Pi = \{\Pi_1, \ldots, \Pi_m\}$ be a PVM and let $M = \{M_1, \ldots, M_n\}$ be an arbitrary POVM. It is known that Π and M are compatible if and only if each of the Jordan product operators $\Pi_i \odot M_j$ is positive semidefinite [12]. In this section, we discuss a similar result for channels.

For the PVM $\Pi = \{\Pi_1, \ldots, \Pi_m\}$, consider the measurement channel $\Delta_\Pi \in \mathrm{C}(\mathcal{X}, \mathcal{Z})$ defined as

$$\Delta_\Pi(X) = \sum_{i=1}^{m} \langle \Pi_i, X \rangle E_{i,i} \tag{71}$$

for every $X \in \mathrm{L}(\mathcal{X})$, where $\dim(\mathcal{Z}) = m$. Theorem 15 provides necessary and sufficient criteria for a given channel $\Phi \in \mathrm{C}(\mathcal{X}, \mathcal{Y})$ to be compatible with Δ_Π in terms of their Jordan product.

Theorem 15. Let $\{\Pi_1, \ldots, \Pi_m\}$ be a PVM. Define the channels $\Xi_\Pi \in \mathrm{C}(\mathcal{X})$ and $\Delta_\Pi \in \mathrm{C}(\mathcal{X})$ as

$$\Xi_\Pi(X) = \sum_{i=1}^{m} \Pi_i X \Pi_i \qquad \text{and} \qquad \Delta_\Pi(X) = \sum_{i=1}^{m} \langle \Pi_i, X \rangle E_{i,i} \tag{72}$$

for every $X \in \mathrm{L}(\mathcal{X})$, and let $\Phi \in \mathrm{C}(\mathcal{X}, \mathcal{Y})$ be a channel. The following are equivalent.

(1) The map $\Phi \odot \Delta_\Pi$ is completely positive.

(2) The channels Φ and Δ_Π are compatible.

(3) It holds that $\Phi = \Phi \circ \Xi_\Pi$.

Note that the map Ξ_Π as defined in Theorem 15 is called the pinching channel corresponding to the PVM Π.

Proof. The implication (1) $\Longrightarrow$ (2) follows from Corollary 13. To prove the implication (2) $\Longrightarrow$ (3), suppose that the channels Φ and Δ_Π are compatible. By Proposition 7, there exist completely positive maps $\Phi_1, \ldots, \Phi_m \in \mathrm{T}(\mathcal{X}, \mathcal{Y})$ such that $\Phi = \sum_{i=1}^{m} \Phi_i$ and satisfy $\mathrm{Tr}_\mathcal{Y}(J(\Phi_i)) = \Pi_i$ for each index $i \in \{1, \ldots, m\}$. It follows from Lemma 5, for each index $i \in \{1, \ldots, m\}$, that

$$J(\Phi_i) = (\Pi_i \otimes \mathbb{1}_\mathcal{Y}) J(\Phi_i) (\Pi_i \otimes \mathbb{1}_\mathcal{Y}) \tag{73}$$

since $\Pi_{\mathrm{im}(\mathrm{Tr}_\mathcal{Y}(J(\Phi_i)))} = \Pi_i$, and thus

$$\Phi_i(X) = \Phi_i(\Pi_i X \Pi_i) \tag{74}$$

holds for every $X \in \mathrm{L}(\mathcal{X})$. Making use of the equality in Eq. (74), and the fact that $\Pi_i \Pi_j = 0$ whenever $i \neq j$, we have that

$$\begin{aligned} (\Phi \circ \Xi_\Pi)(X) &= \sum_{i=1}^{m} \sum_{j=1}^{m} \Phi_i(\Pi_j X \Pi_j) \\ &= \sum_{i=1}^{m} \sum_{j=1}^{m} \Phi_i(\Pi_i \Pi_j X \Pi_j \Pi_i) \\ &= \sum_{i=1}^{m} \Phi_i(\Pi_i X \Pi_i) \\ &= \sum_{i=1}^{m} \Phi_i(X) \\ &= \Phi(X) \end{aligned} \tag{75}$$

for every $X \in \mathrm{L}(\mathcal{X})$, and thus $\Phi = \Phi \circ \Xi_\Pi$.

Finally, to prove the implication (3) $\Longrightarrow$ (1), suppose that $\Phi = \Phi \circ \Xi_\Pi$. Note that the channels Ξ_Π and Δ_Π are compatible. Indeed, it may be verified that the Jordan product of these channels can be expressed as

$$(\Xi_\Pi \odot \Delta_\Pi)(X) = \sum_{i=1}^{m} \Pi_i X \Pi_i \otimes E_{i,i}. \tag{76}$$

This map is clearly completely positive and therefore compatibilizes Ξ_Π and Δ_Π. Hence the map

$$\Phi \circ \Delta_\Pi = (\Phi \circ \Xi_\Pi) \odot \Delta_\Pi = (\Phi \otimes \mathbb{1}_{\mathrm{L}(\mathcal{Z})}) \circ (\Xi_\Pi \circ \Delta_\Pi) \tag{77}$$

is completely positive, as it is the composition of completely positive maps. This completes the proof.

If $\Pi = \{E_{1,1}, \ldots, E_{n,n}\}$ is the PVM consisting of the rank-one projections in the computation basis (where $n = \dim(\mathcal{X})$), the channel $\Delta_\Pi = \Delta$ is simply the completely dephasing channel on $\mathcal{X}$,

$$\Delta(X) = \sum_{i=1}^{n} \langle E_{i,i}, X \rangle E_{i,i}. \tag{78}$$

The map Δ is the measurement channel corresponding to measuring in the computational basis. Applying the result of Theorem 15 in this case provides us with the following necessary and sufficient conditions for a channel to be compatible with the completely dephasing channel Δ.

Corollary 16. Let $\Phi \in \mathrm{C}(\mathcal{X}, \mathcal{Y})$ be a channel and let $\Delta \in \mathrm{C}(\mathcal{X})$ be the completely dephasing channel. The following are equivalent.

(1) The map $\Phi \odot \Delta$ is completely positive.

(2) The channels Φ and Δ are compatible.

(3) It holds that Φ is a measure-and-prepare channel generated by the computational basis PVM.

Proof. Let $n = \dim(\mathcal{X})$. For the PVM $\Pi = \{E_{1,1}, \ldots, E_{n,n}\}$, it is evident that $\Xi_\Pi = \Delta_\Pi = \Delta$, where Ξ_Π and Δ_Π are the channels as defined in Eq. (72). By Theorem 15, it will suffice to show that the condition in (3) is equivalent to the condition that $\Phi = \Phi \circ \Delta$. If $\Phi \circ \Delta = \Phi$ then

$$\Phi(X) = \Phi(\Delta(X)) = \sum_{i=1}^{n} \langle E_{i,i}, X \rangle \Phi(E_{i,i}) = \sum_{i=1}^{n} \langle E_{i,i}, X \rangle \rho_i, \tag{79}$$

for every $X \in \mathrm{L}(\mathcal{X})$, where $\rho_1, \ldots, \rho_n \in \mathrm{D}(\mathcal{Y})$ are the density matrices defined as $\rho_i = \Phi(E_{i,i})$ for each i, and thus Φ satisfies condition (3). On the other hand, if Φ satisfies condition (3) then there exist density matrices $\rho_1, \ldots, \rho_n$ such that Φ may be expressed as

$$\Phi(X) = \sum_{i=1}^{n} \langle E_{i,i}, X \rangle \rho_i, \tag{80}$$

and thus

$$\Phi(\Delta(X)) = \sum_{i=1}^{n} \sum_{j=1}^{n} \langle E_{i,i}, \langle E_{j,j}, X \rangle E_{j,j} \rangle \rho_i = \sum_{i=1}^{n} \langle E_{i,i}, X \rangle \rho_i = \Phi(X) \tag{81}$$

holds for every $X \in \mathrm{L}(\mathcal{X})$.

For the sake of completeness we also investigate other extreme cases.

Lemma 17. Let $\mathcal{X}$ and let $\Phi_U \in \mathrm{C}(\mathcal{X})$ be a unitary channel, i.e., $\Phi_U(X) = UXU^*$ for some $U \in \mathrm{U}(\mathcal{X})$. Let $\Psi \in \mathrm{C}(\mathcal{X}, \mathcal{Y})$. Then the following are equivalent.

(1) Φ_U and Ψ are compatible.

(2) Ψ is a constant channel, i.e., there is a fixed state $\rho \in \mathrm{D}(\mathcal{Y})$ such that $\Psi(X) = \mathrm{Tr}(X)\rho$ for all $X \in \mathrm{L}(\mathcal{X})$.

(3) $J(\Phi_U \odot \Psi) \geq 0$.

Proof. To prove the implication (1) $\Longrightarrow$ (2), note that $J(\Phi_U)$ is a multiple of a maximally entangled state. Suppose $\Phi \in \mathrm{C}(\mathcal{X}, \mathcal{X}' \otimes \mathcal{Y})$ is a compatibilizer of Φ_U and Ψ, where, for convenience, we define $\mathcal{X}' = \mathcal{X}$. It follows that $\mathrm{Tr}_{\mathcal{Y}}(J(\Phi)) = J(\Phi_U)$. By the monogamy of entanglement, it holds that

$$J(\Phi) = J(\Phi_U) \otimes Y. \tag{82}$$

for some fixed choice of positive operator $Y \in \mathrm{Pos}(\mathcal{Y})$. Now,

$$J(\Psi) = \mathrm{Tr}_{\mathcal{X}'}(J(\Phi)) = \mathrm{Tr}_{\mathcal{X}'}(J(\Phi_U) \otimes Y) = \mathbb{1}_{\mathcal{X}} \otimes Y. \tag{83}$$

For all $X \in \mathrm{L}(\mathcal{X})$, one has that

$$\Psi(X) = \mathrm{Tr}_{\mathcal{X}}((X^T \otimes \mathbb{1}_{\mathcal{Y}})J(\Psi)) = \mathrm{Tr}_{\mathcal{X}}((X^T \otimes Y) = \mathrm{Tr}(X)Y. \tag{84}$$

The fact that $\mathrm{Tr}(Y) = 1$ (and thus Y is a state) follows from the fact that Ψ must be trace preserving.

To prove the implication (2) $\Longrightarrow$ (3), let $\rho \in \mathrm{D}(\mathcal{Y})$ be a state such that $\Psi(X) = \mathrm{Tr}(X)\rho$ for every $X \in \mathrm{L}(\mathcal{X})$. Note that

$$J(\Phi_U \odot \Psi) = J(\Phi_U) \otimes \rho, \tag{85}$$

and thus the operator $J(\Phi_U \odot \Psi)$ is clearly positive semidefinite. The implication (3) $\Longrightarrow$ (1) follows from Corollary 13.

Lemma 18. Let $\Phi_\rho \in \mathrm{C}(\mathcal{X}, \mathcal{Y})$ be a constant channel, i.e., $\Phi_\rho(X) = \mathrm{Tr}(X)\rho$ for all $X \in \mathrm{L}(X)$, for some fixed choice of state $\rho \in \mathrm{D}(\mathcal{Y})$. Let $\Psi \in \mathrm{C}(\mathcal{X}, \mathcal{Z})$. It holds that $J(\Phi_\rho \odot \Psi) \geq 0$ and the channels Φ_ρ and Ψ are compatible.

Proof. As in the proof of Lemma 17, it is evident that

$$J(\Psi \odot \Phi_\rho) = J(\Psi) \otimes \rho, \tag{86}$$

where we have changed the order of the channels for convenience.

4.3 Generalized Jordan products

One may ask whether the Jordan product of maps given by Definition 11 is the unique construction that satisfies the key properties outlined in Proposition 12 and Corollary 13. We now show that this is not the case by generalizing the construction of the Jordan product of linear maps.

Definition 19 (Generalized Jordan product of linear maps). Let $\mathcal{X} = \mathcal{X}_1 = \mathcal{X}_2$ be complex Euclidean spaces and let $A \in \mathrm{Herm}(\mathcal{X} \otimes \mathcal{X}_1 \otimes \mathcal{X}_2)$ be an operator satisfying

$$\mathrm{Tr}_{\mathcal{X}_1}(A) = \mathrm{Tr}_{\mathcal{X}_2}(A) = \sum_{i,j=1}^{\dim(\mathcal{X})} E_{i,j} \otimes E_{i,j}. \tag{87}$$

Let $\Phi_1 \in \mathrm{T}(\mathcal{X}, \mathcal{Y}_1)$ and $\Phi_2 \in \mathrm{T}(\mathcal{X}, \mathcal{Y}_2)$ be linear maps. The generalized Jordan product of the maps Φ_1 and Φ_2 with respect to the operator A is the linear map

$$\Phi_1 \odot_A \Phi_2 \in \mathrm{T}(\mathcal{X}, \mathcal{Y}_1 \otimes \mathcal{Y}_2) \tag{88}$$

whose Choi representation is the operator $J(\Phi_1 \odot_A \Phi_2) \in \mathrm{L}(\mathcal{X} \otimes \mathcal{Y}_1 \otimes \mathcal{Y}_2)$ given by

$$J(\Phi_1 \odot_A \Phi_2) = \big(\mathbb{1}_{\mathrm{L}(\mathcal{X})} \otimes \Phi_1 \otimes \Phi_2\big)(A). \tag{89}$$

Note that the requirement in Eq. (87) can be rephrased as

$$\mathrm{Tr}_{\mathcal{X}_1}(A) = \mathrm{Tr}_{\mathcal{X}_2}(A) = J(\mathbb{1}_{\mathrm{L}(\mathcal{X})}). \tag{90}$$

If one defines the operator $A_{\mathrm{JP}} \in \mathrm{Herm}(\mathcal{X} \otimes \mathcal{X}_1 \otimes \mathcal{X}_2)$ as

$$A_{\mathrm{JP}} = \mathbb{1}_{\mathrm{L}(\mathcal{X})} \odot \mathbb{1}_{\mathrm{L}(\mathcal{X})} = \frac{1}{2} \sum_{i,j=1}^{\dim(\mathcal{X})} E_{i,j} \otimes \left(\sum_{k=1}^{\dim(\mathcal{X})} E_{i,k} \otimes E_{k,j} + E_{k,j} \otimes E_{i,k} \right), \tag{91}$$

one recovers the (standard) Jordan product from Definition 11 by choosing $A = A_{\mathrm{JP}}$. That is, the (standard) Jordan product of linear maps $\Phi_1 \in \mathrm{T}(\mathcal{X}, \mathcal{Y}_1)$ and $\Phi_1 \in \mathrm{T}(\mathcal{X}, \mathcal{Y}_1)$ may be expressed as

$$\Phi_1 \odot \Phi_2 = \Phi_1 \odot_{A_{\mathrm{JP}}} \Phi_2. \tag{92}$$

This choice of A is not unique. Indeed, for any choice of nonzero Hermitian operator $X \in \mathrm{Herm}(\mathcal{X})$ satisfying $\mathrm{Tr}(X) = 0$, one may define

$$A = A_{\mathrm{JP}} + \mathbb{1}_{\mathcal{X}} \otimes X \otimes X. \tag{93}$$

This operator clearly satisfies Eq. (87) and thus defines a generalized Jordan product that is distinct from the standard one $\odot_{A_{\mathrm{JP}}}$.

The generalized Jordan product as defined in Definition 19 possesses all of the key properties outlined in Proposition 12 and Corollary 13 that are satisfied by the standard Jordan product. In particular, for any choice of trace-preserving linear maps $\Phi_1 \in \mathrm{T}(\mathcal{X}, \mathcal{Y}_1)$ and $\Phi_2 \in \mathrm{T}(\mathcal{X}, \mathcal{Y}_2)$, one has

$$\mathrm{Tr}_{\mathcal{Y}_2}(J(\Phi_1 \odot_A \Phi_2)) = J(\Phi_1) \qquad \text{and} \qquad \mathrm{Tr}_{\mathcal{Y}_1}(J(\Phi_1 \odot_A \Phi_2)) = J(\Phi_2) \tag{94}$$

and, moreover, the map $\Phi_1 \odot_A \Phi_2$ is also trace preserving when Φ_1 and Φ_2 are trace preserving. In other words, Proposition 12 holds for generalized Jordan products as well. Indeed, making use of the assumption that Φ_2 is trace preserving, one has that

$$\mathrm{Tr}_{\mathcal{Y}_2}(J(\Phi_1 \odot_A \Phi_2)) = (\mathbb{1}_{\mathrm{L}(\mathcal{X})} \otimes \Phi_1)\big(\mathrm{Tr}_{\mathcal{Y}_2}(A)\big) = (\mathbb{1}_{\mathrm{L}(\mathcal{X})} \otimes \Phi_1)(J(\mathbb{1}_{\mathrm{L}(\mathcal{X})})) = J(\Phi_1). \tag{95}$$

An analogous argument shows that $\mathrm{Tr}_{\mathcal{Y}_1}(J(\Phi_1 \odot_A \Phi_2)) = J(\Phi_2)$ which follows from the assumption that Φ_1 is trace preserving. Moreover, if Φ_1 and Φ_2 are Hermitian-preserving, then $\Phi_1 \odot_A \Phi_2$ is also Hermitian-preserving.

Analogous to the (standard) Jordan product, a generalized Jordan product provides a useful condition to check to see if two channels are compatible.

Proposition 20. Let $\Phi_1 \in C(\mathcal{X}, \mathcal{Y}_1)$ and $\Phi_2 \in C(\mathcal{X}, \mathcal{Y}_2)$ be two quantum channels. If there exists $A \in \mathrm{Herm}(\mathcal{X} \otimes \mathcal{X}_1 \otimes \mathcal{X}_2)$, where $\mathcal{X} = \mathcal{X}_1 = \mathcal{X}_2$, satisfying Eq. (87) such that $\Phi_1 \odot_A \Phi_2$ is completely positive, then $\Phi_1 \odot_A \Phi_2$ compatibilizes Φ_1 and Φ_2.

Proof. This follows directly from observations in the previous paragraph.

This suggests the following definition.

Definition 21 (Jordan compatible). We say that $\Phi_1 \in C(\mathcal{X}, \mathcal{Y}_1)$ and $\Phi_2 \in C(\mathcal{X}, \mathcal{Y}_2)$ are Jordan compatible if there exists an operator $A \in \mathrm{Herm}(\mathcal{X} \otimes \mathcal{X}_1 \otimes \mathcal{X}_2)$ satisfying Eq. (87) (where $\mathcal{X} = \mathcal{X}_1 = \mathcal{X}_2$) such that $\Phi_1 \odot_A \Phi_2$ is completely positive.

Therefore, if two channels are Jordan compatible, then they are compatible.

Remark 22. In this work, we consider both the generalized Jordan product and the standard version. We sometimes refer to (standard) Jordan compatibility or (generalized) Jordan compatibility to emphasize which one we mean.

4.4 Generalizing Jordan products of matrices

Following the procedure of generalizing the Jordan product for linear maps, one may use similar ideas to generalize the Jordan product of operators in the following manner. For every Hermitian operator $A \in \mathrm{Herm}(\mathcal{X} \otimes \mathcal{X} \otimes \mathcal{X})$ having the form $A = A_{\mathrm{JP}} + \mathbb{1}_{\mathcal{X}} \otimes X \otimes X$ for some fixed choice of Hermitian operator $X \in \mathrm{Herm}(\mathcal{X})$ satisfying $\mathrm{Tr}(X) = 0$, one may define the generalized Jordan product of operators with respect to A as

$$B \odot_A C = B \odot C + \langle X \otimes X, B \otimes C \rangle \mathbb{1}_{\mathcal{X}}. \tag{96}$$

This type of generalized Jordan product of operators provides a condition for checking if two POVMs are compatible. Suppose $\{M_1, \ldots, M_m\} \subset \mathrm{Pos}(\mathcal{X})$ and $\{N_1, \ldots, N_n\} \subset \mathrm{Pos}(\mathcal{X})$ are POVMs. If it is the case that

$$M_i \odot_A N_j \geq 0 \tag{97}$$

for each pair of indices i and j (where $\odot_A$ is the generalized Jordan product as defined in Eq. (96)), then the POVMs M and N are compatible as the operators defined as $P_{i,j} = M_i \odot N_j$ necessarily compose a compatibilizing POVM.

4.5 Jordan product compatibility of channels

It is natural to ask if the converse to the main result of the previous subsection also holds. That is, if two channels are compatible, are they necessarily Jordan compatible? The results of Proposition 16, Proposition 15, Lemma 17 and Lemma 18 show that this is true if either channel is of a certain type. We now show that this is also true for other classes of channels as well.

In the following, we make use of the inverse map (if it exists) of a linear map of the form $\Phi \in T(\mathcal{X}, \mathcal{Y})$. If Φ is completely positive and invertible as a linear map, its inverse Φ^{-1} may not necessarily be completely positive. However, the following lemma shows that Φ^{-1} is necessarily trace preserving if Φ is trace preserving.

Note the following: let $\Phi \in T(\mathcal{X}, \mathcal{Y})$ and assume that an inverse map $\Phi^{-1} \in T(\mathcal{Y}, \mathcal{X})$ exists. Then the vector spaces $L(\mathcal{X})$ and $L(\mathcal{Y})$ are isomorphic and so also $\mathcal{X}$ and $\mathcal{Y}$ are isomorphic.

Lemma 23. Let $\Phi \in \mathrm{T}(\mathcal{X}, \mathcal{Y})$ be an invertible linear map. The map Φ is trace preserving if and only if its inverse map $\Phi^{-1} \in \mathrm{T}(\mathcal{Y}, \mathcal{X})$ is trace preserving.

Proof. Suppose that Φ is trace preserving. For every $Y \in \mathrm{L}(\mathcal{Y})$, one has that

$$\mathrm{Tr}(\Phi^{-1}(Y)) = \mathrm{Tr}(\Phi(\Phi^{-1}(Y))) = \mathrm{Tr}(Y), \tag{98}$$

where the first equality follows from the assumption that Φ is trace preserving. The rest of the proof follows by symmetry between Φ and Φ^{-1}.

We now state the equivalence of channel compatibility and Jordan compatibility for certain pairs of channels.

Theorem 24. Let $\Phi_1 \in \mathrm{C}(\mathcal{X}, \mathcal{Y}_1)$ and $\Phi_2 \in \mathrm{C}(\mathcal{X}, \mathcal{Y}_2)$ be two quantum channels, such that they have inverse linear maps $\Phi_1^{-1} \in \mathrm{T}(\mathcal{Y}_1, \mathcal{X})$ and $\Phi_2^{-1} \in \mathrm{T}(\mathcal{Y}_2, \mathcal{X})$. The channels Φ_1 and Φ_2 are compatible if and only if they are Jordan compatible.

Proof. By Proposition 20, if the channels are Jordan compatible then they are compatible. To prove the converse, assume the channels are compatible and let $\Phi \in \mathrm{C}(\mathcal{X}, \mathcal{Y}_1 \otimes \mathcal{Y}_2)$ be a compatibilizing channel. Define the operator $A \in \mathrm{Herm}(\mathcal{X} \otimes \mathcal{X}_1 \otimes \mathcal{X}_2)$, where $\mathcal{X} = \mathcal{X}_1 = \mathcal{X}_2$, as

$$A = J\big((\Phi_1^{-1} \otimes \Phi_2^{-1}) \circ \Phi\big). \tag{99}$$

We now show that A satisfies Eq. (87). Note that

$$\begin{aligned} \mathrm{Tr}_{\mathcal{X}_1}(A) &= J\big(((\mathrm{Tr} \circ \Phi_1^{-1}) \otimes \Phi_2^{-1}) \circ \Phi\big) \\ &= J\big((\mathrm{Tr} \otimes \Phi_2^{-1}) \circ \Phi\big) \\ &= J\big(\Phi_2^{-1} \circ (\mathrm{Tr}_{\mathcal{Y}_1} \circ \Phi)\big) \\ &= J\big(\Phi_2^{-1} \circ \Phi_2\big) \\ &= J(\mathbb{1}_{\mathrm{L}(\mathcal{X})}), \end{aligned} \tag{100}$$

where equality in the second line follows from the fact that Φ_1^{-1} is trace preserving by Lemma 23. Similarly, one finds that

$$\mathrm{Tr}_{\mathcal{X}_2}(A) = J\big(\Phi_1^{-1} \otimes \mathrm{Tr}) \circ \Phi\big) = J(\Phi_1^{-1} \circ \Phi_1) = J(\mathbb{1}_{\mathrm{L}(\mathcal{X})}), \tag{101}$$

which completes the proof. Note that $J(\Phi_1 \odot_A \Phi_2) \geq 0$ simply because

$$J(\Phi_1 \odot_A \Phi_2) = (\mathbb{1}_{\mathrm{L}(\mathcal{X})} \otimes \Phi_1 \otimes \Phi_2)(A) = (\mathbb{1}_{\mathrm{L}(\mathcal{X})} \otimes \Phi_1 \otimes \Phi_2)(J\big((\Phi_1^{-1} \otimes \Phi_2^{-1}) \circ \Phi\big)) = J(\Phi). \tag{102}$$

Almost all linear maps in $\mathrm{T}(\mathcal{X})$ are invertible. Hence naive numerical approaches cannot be used to search for a pair of channels $\Phi_1, \Phi_2 \in \mathrm{C}(\mathcal{X})$ that are compatible but not Jordan compatible (if such a pair exists), as this would involve randomly sampling from a set having zero measure. For this reason, the question of whether compatibility of channels is equivalent to Jordan compatibility remains open. We conjecture that such a pair of channels does not exists. Nonetheless, it can be shown that the set of Jordan-compatible pairs of channels is dense in the set of all pairs of compatible channels.

Supplementary Note 5 - Geometry of pairs of compatible channels

In this section, we discuss the geometry of the set of compatible pairs of channels. To this end, we introduce some notation. Let $\mathrm{HP}(\mathcal{X},\mathcal{Y})$ denote the space of Hermitian-preserving linear maps from $\mathrm{L}(\mathcal{X})$ to $\mathrm{L}(\mathcal{Y})$. This is a real vector space with dimension

$$\dim\big(\mathrm{HP}(\mathcal{X},\mathcal{Y})\big) = \dim(\mathcal{X})^2 \dim(\mathcal{Y})^2. \tag{103}$$

Here we are concerned with the space $\mathrm{HP}(\mathcal{X},\mathcal{Y}_1) \oplus \mathrm{HP}(\mathcal{X},\mathcal{Y}_2)$ of pairs of such linear maps.

Definition 25. We define the following sets of pairs of Hermitian-preserving linear maps:

- $\mathrm{CPairs}(\mathcal{X},\mathcal{Y}_1,\mathcal{Y}_2) = \{(\Phi_1,\Phi_2) : \Phi_1 \in \mathrm{C}(\mathcal{X},\mathcal{Y}_1),\ \Phi_2 \in \mathrm{C}(\mathcal{X},\mathcal{Y}_2)\}$
- $\mathrm{Comp}(\mathcal{X},\mathcal{Y}_1,\mathcal{Y}_2) = \{(\Phi_1,\Phi_2) \in \mathrm{CPairs} : \Phi_1 \text{ and } \Phi_2 \text{ are compatible}\}$
- $\mathrm{JComp}(\mathcal{X},\mathcal{Y}_1,\mathcal{Y}_2) = \{(\Phi_1,\Phi_2) \in \mathrm{CPairs} : \Phi_1 \text{ and } \Phi_2 \text{ are Jordan compatible}\}$.

It is evident that we have the containments

$$\mathrm{JComp}(\mathcal{X},\mathcal{Y}_1,\mathcal{Y}_2) \subseteq \mathrm{Comp}(\mathcal{X},\mathcal{Y}_1,\mathcal{Y}_2) \subsetneq \mathrm{CPairs}(\mathcal{X},\mathcal{Y}_1,\mathcal{Y}_2). \tag{104}$$

The remainder of this section is dedicated to stating and proving a few facts regarding the geometry of these sets, which we summarize here to outline our approach:

1. The set $\mathrm{Comp}(\mathcal{X},\mathcal{Y}_1,\mathcal{Y}_2)$ is compact and convex.
2. The set $\mathrm{Comp}(\mathcal{X},\mathcal{Y}_1,\mathcal{Y}_2)$ has positive measure as a subset of $\mathrm{CPairs}(\mathcal{X},\mathcal{Y}_1,\mathcal{Y}_2)$. (That is, a randomly selected pair of channels has a nonzero probability of being compatible.)
3. In the case when $\mathcal{X} = \mathcal{Y}_1 = \mathcal{Y}_2$, almost all pairs of compatible channels are Jordan compatible. (That is, the set of non-Jordan-compatible pairs has zero measure as a subset of $\mathrm{Comp}(\mathcal{X},\mathcal{X},\mathcal{X})$).
4. In particular, one has that $\overline{\mathrm{JComp}(\mathcal{X},\mathcal{X},\mathcal{X})} = \mathrm{Comp}(\mathcal{X},\mathcal{X},\mathcal{X})$ (where $\overline{\mathcal{A}}$ indicates the topological closure of a set $\mathcal{A}$).

We stress that the above implies that with probability 1, a randomly selected pair of compatible channels (with respect to the measure as discussed below) is also Jordan compatible. This rules out, for instance, a brute-force random search to find a pair of channels that are compatible but not Jordan compatible.

Since we are referring to probabilities and measure-zero sets of pairs of channels, it is necessary to clarify the measure on the set $\mathrm{CPairs}(\mathcal{X},\mathcal{Y}_1,\mathcal{Y}_2)$. Note that $\mathrm{CPairs}(\mathcal{X},\mathcal{Y}_1,\mathcal{Y}_2)$ is a compact and convex subset of the affine subspace of pairs of trace-preserving maps in the Euclidean space $\mathrm{HP}(\mathcal{X},\mathcal{Y}_1) \oplus \mathrm{HP}(\mathcal{X},\mathcal{Y}_2)$. Thus the set CPairs is a submanifold and has a measure that is induced by the natural Lebesgue measure of the underlying Euclidean space. (See, e.g., [13, Section 5.5].)

5.1 Convexity of the set of pairs of compatible channels

Convexity of the set $\text{CPairs}(\mathcal{X}, \mathcal{Y}_1, \mathcal{Y}_2)$ follows trivially from convexity of $\text{C}(\mathcal{X}, \mathcal{Y}_1)$ and $\text{C}(\mathcal{X}, \mathcal{Y}_2)$. Importantly, the set of compatible pairs of channels is also convex—a fact that we prove in the following lemma.

Lemma 26. The set $\text{Comp}(\mathcal{X}, \mathcal{Y}_1, \mathcal{Y}_2)$ is convex.

Proof. Let (Φ_1, Φ_2) and (Ψ_1, Ψ_2) be pairs of compatible channels, let $\Phi, \Psi \in \text{C}(\mathcal{X}, \mathcal{Y}_1 \otimes \mathcal{Y}_2)$ be respective channels that compatibilize these pairs, and let $\lambda \in [0,1]$. One has that

$$\text{Tr}_{\mathcal{Y}_2} \circ (\lambda\Phi + (1-\lambda)\Psi) = \lambda \, \text{Tr}_{\mathcal{Y}_2} \circ \Phi + (1-\lambda) \, \text{Tr}_{\mathcal{Y}_2} \circ \Psi = \lambda\Phi_1 + (1-\lambda)\Psi_1 \tag{105}$$

and, analogously, that $\text{Tr}_{\mathcal{Y}_1} \circ (\lambda\Phi + (1-\lambda)\Psi) = \lambda\Phi_2 + (1-\lambda)\Psi_2$. It follows that $\lambda\Phi + (1-\lambda)\Psi$ is a channel that compatibilizes the pair

$$\lambda(\Phi_1, \Phi_2) + (1-\lambda)(\Psi_1, \Psi_2) = \big(\lambda\Phi_1 + (1-\lambda)\Psi_1, \, \lambda\Phi_2 + (1-\lambda)\Psi_2\big) \tag{106}$$

and thus this pair is compatible.

We now prove the following useful result, which states that mixing any pair of channels with a pair of constant channels yields a compatible pair.

Proposition 27. Let $\rho_1 \in \text{D}(\mathcal{Y}_1)$ and $\rho_2 \in \text{D}(\mathcal{Y}_2)$ be states and let $\Phi_{\rho_1} \in \text{C}(\mathcal{X}, \mathcal{Y}_1)$ and $\Phi_{\rho_2} \in \text{C}(\mathcal{X}, \mathcal{Y}_2)$ be the constant channels defined as

$$\Phi_{\rho_1}(X) = \text{Tr}(X)\rho_1 \qquad \text{and} \qquad \Phi_{\rho_2}(X) = \text{Tr}(X)\rho_2 \tag{107}$$

for every $X \in \text{L}(\mathcal{X})$. For every other pair of channels $\Psi_1 \in \text{C}(\mathcal{X}, \mathcal{Y}_1)$ and $\Psi_2 \in \text{C}(\mathcal{X}, \mathcal{Y}_2)$, it holds that

$$\left(\frac{1}{2}\Psi_1 + \frac{1}{2}\Phi_{\rho_1}, \, \frac{1}{2}\Psi_2 + \frac{1}{2}\Phi_{\rho_2}\right) \in \text{Comp}(\mathcal{X}, \mathcal{Y}_1, \mathcal{Y}_2). \tag{108}$$

Proof. It is evident that the constant channels Φ_{ρ_1} and Φ_{ρ_2} are each compatible with every channel in $\text{C}(\mathcal{X}, \mathcal{Y}_2)$ and $\text{C}(\mathcal{X}, \mathcal{Y}_1)$ (see Lemma 18). In particular, one has that

$$(\Psi_1, \Phi_{\rho_2}) \in \text{Comp}(\mathcal{X}, \mathcal{Y}_1, \mathcal{Y}_2) \qquad \text{and} \qquad (\Phi_{\rho_1}, \Psi_2) \in \text{Comp}(\mathcal{X}, \mathcal{Y}_1, \mathcal{Y}_2). \tag{109}$$

It follows from convexity (Lemma 26) that the pair

$$\frac{1}{2}(\Psi_1, \Phi_{\rho_2}) + \frac{1}{2}(\Phi_{\rho_1}, \Psi_2) = \left(\frac{1}{2}\Psi_1 + \frac{1}{2}\Phi_{\rho_1}, \, \frac{1}{2}\Psi_2 + \frac{1}{2}\Phi_{\rho_2}\right) \tag{110}$$

is compatible.

Importantly, we also point out that the set of compatible pairs is also closed, as proved below.

Proposition 28. The set $\text{Comp}(\mathcal{X}, \mathcal{Y}_1, \mathcal{Y}_2)$ is compact.

Proof. This may be proved by observing that $\text{Comp}(\mathcal{X}, \mathcal{Y}_1, \mathcal{Y}_2)$ is the image of the set of channels $\text{C}(\mathcal{X}, \mathcal{Y}_1 \otimes \mathcal{Y}_2)$ under the linear map $\text{HP}(\mathcal{X}, \mathcal{Y}_1 \otimes \mathcal{Y}_2) \to \text{HP}(\mathcal{X}, \mathcal{Y}_1) \oplus \text{HP}(\mathcal{X}, \mathcal{Y}_2)$ that is defined by $\Phi \mapsto (\text{Tr}_{\mathcal{Y}_2} \circ \Phi, \text{Tr}_{\mathcal{Y}_1} \circ \Phi)$. The desired result follows from the fact that $\text{C}(\mathcal{X}, \mathcal{Y}_1 \otimes \mathcal{Y}_2)$ is compact.

5.2 Norm and measure for pairs of channels

We may define a norm on the real vector space $\mathrm{HP}(\mathcal{X},\mathcal{Y}_1) \oplus \mathrm{HP}(\mathcal{X},\mathcal{Y}_2)$ as follows. For a pair (Φ_1,Φ_2) of Hermitian-preserving maps, define

$$\|(\Phi_1,\Phi_2)\|_J = \|J(\Phi_1)\| + \|J(\Phi_2)\| \tag{111}$$

where $\|J(\Phi_1)\|$ and $\|J(\Phi_2)\|$ denote the operator norms of the Choi representations of Φ_1 and Φ_2. The following lemma shows the existence of a ball of positive radius that is fully contained in the set of pairs of channels.

Lemma 29. Let $\Omega_{\mathcal{Y}_1} \in \mathrm{C}(\mathcal{X},\mathcal{Y}_1)$ and $\Omega_{\mathcal{Y}_2} \in \mathrm{C}(\mathcal{X},\mathcal{Y}_2)$ be the constant channels defined as

$$\Omega_{\mathcal{Y}_1}(X) = \frac{\mathrm{Tr}(X)}{\dim(\mathcal{Y}_1)}\mathbb{1}_{\mathcal{Y}_1} \qquad \text{and} \qquad \Omega_{\mathcal{Y}_2}(X) = \frac{\mathrm{Tr}(X)}{\dim(\mathcal{Y}_2)}\mathbb{1}_{\mathcal{Y}_2} \tag{112}$$

for all $X \in \mathrm{L}(\mathcal{X})$ and let $(\Phi_1,\Phi_2) \in \mathrm{HP}(\mathcal{X},\mathcal{Y}_1) \oplus \mathrm{HP}(\mathcal{X},\mathcal{Y}_1)$ be a pair of Hermitian-preserving maps for which

$$\left\|(\Phi_1,\Phi_2) - (\Omega_{\mathcal{Y}_1},\Omega_{\mathcal{Y}_2})\right\|_J \leq \min\left\{\frac{1}{\dim(\mathcal{Y}_1)},\frac{1}{\dim(\mathcal{Y}_2)}\right\}. \tag{113}$$

If Φ_1 and Φ_2 are also trace preserving, then $(\Phi_1,\Phi_2) \in \mathrm{CPairs}(\mathcal{X},\mathcal{Y}_1,\mathcal{Y}_2)$.

Proof. Suppose that the Hermitian-preserving linear maps Φ_1 and Φ_2 are trace preserving. It suffices to show that Φ_1 and Φ_2 are completely positive. Note that the inequality in Eq. (113) implies that

$$\|J(\Phi_1) - J(\Omega_{\mathcal{Y}_1})\| \leq \frac{1}{\dim(\mathcal{Y}_1)} \qquad \text{and} \qquad \|J(\Phi_2) - J(\Omega_{\mathcal{Y}_2})\| \leq \frac{1}{\dim(\mathcal{Y}_2)}. \tag{114}$$

Observe that

$$J(\Omega_{\mathcal{Y}_1}) = \frac{1}{\dim(\mathcal{Y}_1)}\mathbb{1}_{\mathcal{X}} \otimes \mathbb{1}_{\mathcal{Y}_1} \qquad \text{and} \qquad J(\Omega_{\mathcal{Y}_1}) = \frac{1}{\dim(\mathcal{Y}_2)}\mathbb{1}_{\mathcal{X}} \otimes \mathbb{1}_{\mathcal{Y}_2}. \tag{115}$$

The inequalities in Eq. (114), together with the equalities in Eq. (115), imply that $J(\Phi_1) \geq 0$ and $J(\Phi_2) \geq 0$, and thus Φ_1 and Φ_2 are completely positive, as required.

For the channels $\Omega_{\mathcal{Y}_1}$ and $\Omega_{\mathcal{Y}_2}$ defined in Eq. (112), Lemma 29 implies that, for any pair (Φ_1,Φ_2) of trace-preserving linear maps that is within a distance of $\min\{1/\dim(\mathcal{Y}_1), 1/\dim(\mathcal{Y}_2)\}$ from the pair of channels $(\Omega_{\mathcal{Y}_1},\Omega_{\mathcal{Y}_2})$, the maps Φ_1 and Φ_2 are also channels. This fact, together with the result from Proposition 27, implies the following proposition.

Proposition 30. Let $\Phi_1 \in \mathrm{C}(\mathcal{X},\mathcal{Y}_1)$ and $\Phi_2 \in \mathrm{C}(\mathcal{X},\mathcal{Y}_2)$ be channels, let $\Omega_{\mathcal{Y}_1}$ and $\Omega_{\mathcal{Y}_2}$ be the channels as defined in Eq. (112), and suppose that

$$\|(\Phi_1,\Phi_2) - (\Omega_{\mathcal{Y}_1},\Omega_{\mathcal{Y}_2})\|_J \leq \frac{1}{2}\min\left\{\frac{1}{\dim(\mathcal{Y}_1)},\frac{1}{\dim(\mathcal{Y}_2)}\right\}. \tag{116}$$

Then the channels Φ_1 and Φ_2 are compatible.

Proof. Consider the linear maps defined as $\Psi_1 = 2\Phi_1 - \Omega_{\mathcal{Y}_2}$ and $\Psi_2 = 2\Phi_2 - \Omega_{\mathcal{Y}_2}$. It is evident that these maps are trace-preserving. We have that

$$\|(\Psi_1, \Psi_2) - (\Omega_{\mathcal{Y}_1}, \Omega_{\mathcal{Y}_2})\|_J = 2\|(\Phi_1, \Phi_2) - (\Omega_{\mathcal{Y}_1}, \Omega_{\mathcal{Y}_2})\|_J \le \min\left\{\frac{1}{\dim(\mathcal{Y}_1)}, \frac{1}{\dim(\mathcal{Y}_2)}\right\}, \tag{117}$$

and thus Ψ_1 and Ψ_2 are channels by Lemma 29. It follows from Proposition 27 that the pair

$$(\Phi_1, \Phi_2) = \frac{1}{2}(\Psi_1, \Psi_2) + \frac{1}{2}(\Omega_{\mathcal{Y}_1}, \Omega_{\mathcal{Y}_2}) \tag{118}$$

is compatible, as desired.

The existence of a ball of positive radius within the set of all pairs of compatible channels implies the following corollary.

Corollary 31. The sets $\mathrm{Comp}(\mathcal{X}, \mathcal{Y}_1, \mathcal{Y}_2)$ and $\mathrm{CPairs}(\mathcal{X}, \mathcal{Y}_1, \mathcal{Y}_2)$ have the same dimension as convex sets. In particular, $\mathrm{Comp}(\mathcal{X}, \mathcal{Y}_1, \mathcal{Y}_2)$ has positive measure in $\mathrm{CPairs}(\mathcal{X}, \mathcal{Y}_1, \mathcal{Y}_2)$.

In particular, this means that a randomly selected pair of channels has a nonzero probability of being compatible.

5.3 Invertible maps and Jordan compatibility

Here we prove that almost all pairs of compatible pairs of channels are Jordan compatible. To do so, we first observe that almost all channels are invertible as linear maps (and thus almost all pairs of channels are invertible pairs).

Proposition 32. Almost all channels in $\mathrm{C}(\mathcal{X})$ are invertible as linear maps (in the sense that non-invertible channels form a set of measure zero).

Proof. We may view all Hermitian-preserving maps on $\mathrm{L}(\mathcal{X})$ as linear maps on the real vector space of Hermitian operators $\mathrm{Herm}(\mathcal{X})$. By setting $n = \dim(\mathcal{X})$, we may identify $\mathrm{Herm}(\mathcal{X}) \simeq \mathbb{R}^{n^2}$, and we may identify the space $\mathrm{HP}(\mathcal{X})$ of Hermitian-preserving maps with the space of $n^2 \times n^2$ matrices over $\mathbb{R}$. Moreover, under these identifications, the affine space of trace-preserving maps in $\mathrm{HP}(\mathcal{X})$ corresponds to some affine subspace of $n^2 \times n^2$ matrices. The set $\mathrm{C}(\mathcal{X})$ of all channels may be identified with a convex subset in this affine subspace. Finally, note that the determinant is a polynomial on the n^4-dimensional vector space of $n^2 \times n^2$ matrices over $\mathbb{R}$, and thus the determinant is either constant on this affine subspace or the set of zeroes has measure zero (with respect to the measure on this affine subspace that is induced by the Lebesgue measure on $\mathbb{R}^{n^4}$). A matrix is invertible if and only if its determinant is nonzero. Since the identity channel is certainly invertible, it follows from the above observations that the set of non-invertible channels (as a subset of all channels) has zero measure.

This immediately implies that almost all pairs of channels are invertible.

Corollary 33. For almost all pairs of channels $(\Phi_1, \Phi_2) \in \mathrm{CPairs}(\mathcal{X}, \mathcal{X}, \mathcal{X})$, the channels Φ_1 and Φ_2 are invertible as linear maps. (That is, the set of pairs of channels such that at least one channel is not invertible has zero measure.)

We may now prove the main result, which is that almost all pairs of compatible channels are Jordan compatible. In particular, this means that the set of compatible channels is equal to the closure of the set of Jordan compatible channels.

Theorem 34. The following statements hold.

(1) The set $\mathrm{JComp}(\mathcal{X},\mathcal{X},\mathcal{X})$ has full measure as a subset of $\mathrm{Comp}(\mathcal{X},\mathcal{X},\mathcal{X})$.

(2) $\overline{\mathrm{JComp}(\mathcal{X},\mathcal{X},\mathcal{X})} = \mathrm{Comp}(\mathcal{X},\mathcal{X},\mathcal{X})$.

Proof. Consider the set of pairs of invertible channels, which we denote as

$$\mathrm{InvPairs}(\mathcal{X},\mathcal{X},\mathcal{X}) = \{(\Phi_1,\Phi_2) \in \mathrm{CPairs}(\mathcal{X},\mathcal{X},\mathcal{X}) \; : \; \Phi_1,\Phi_2 \text{ are invertible as linear maps}\}. \tag{119}$$

Recall from Theorem 24 that, a pair of invertible channels $(\Phi_1,\Phi_2) \in \mathrm{InvPairs}(\mathcal{X},\mathcal{X},\mathcal{X})$ is compatible if and only if it is Jordan compatible. It follows that

$$\mathrm{Comp} \cap \mathrm{InvPairs} \subseteq \mathrm{JComp} \subseteq \mathrm{Comp} \tag{120}$$

(where, for simplicity, we have left off the "$(\mathcal{X},\mathcal{X},\mathcal{X})$" part of each set in the above containments). Statement (1) now follows from the facts that Comp has positive measure in CPairs and the set InvPairs has full measure in the set CPairs. Finally, statement (2) is a trivial corollary of statement (1), as the set Comp is closed (see Proposition 28).

We remark that the result of Theorem 34 does not straightforwardly apply to the problem of determining compatibility of measurements and measurement channels. This is because the dimension of the output space of a measurement channel is equal to the number of elements of its underlying POVM, and thus measurement channels are not invertible in general. (A mapping can only be invertible if the input and output dimensions are the same.)

Supplementary Note 6 - Semidefinite programming (SDP) formulations

In this section, we formulate some of the compatibility questions posed in this work as semidefinite programs and examine them in a new light via duality theory. Also, we use semidefinite programming methods to provide a novel proof the well-known result that there is no perfect broadcasting in quantum theory.

6.1 SDP formulations of compatibility

Recall from the introduction that determining whether a given pair of channels $\Phi_1 \in \mathrm{C}(\mathcal{X},\mathcal{Y}_1)$ and $\Phi_2 \in \mathrm{C}(\mathcal{X},\mathcal{Y}_2)$ is compatible is equivalent to solving the following feasibility problem:

$$\begin{aligned} \text{find:} \quad & \Phi \text{ completely positive} \\ \text{satisfying:} \quad & \Phi_1 = \mathrm{Tr}_{\mathcal{Y}_2} \circ \Phi \\ & \Phi_2 = \mathrm{Tr}_{\mathcal{Y}_1} \circ \Phi. \end{aligned} \tag{121}$$

Using the Choi representations, one finds that this is equivalent to the following semidefinite programming feasibility problem:

$$\begin{aligned} \text{find:} \quad & X \in \mathrm{Pos}(\mathcal{X} \otimes \mathcal{Y}_1 \otimes \mathcal{Y}_2) \\ \text{satisfying:} \quad & \mathrm{Tr}_{\mathcal{Y}_2}(X) = J(\Phi_1) \\ & \mathrm{Tr}_{\mathcal{Y}_1}(X) = J(\Phi_2), \end{aligned} \tag{122}$$

where a solution X to the problem Eq. (122) is the Choi representation of a compatibilizing channel in Eq. (121) (if one exists). In other words, we are translating the channel problem into a matrix problem via the Choi isomorphism.

The channel compatibility problem can also be phrased in terms of the following pair of semidefinite programs. Let α_C be the optimal value of the following semidefinite program

$$\begin{aligned} \text{maximize:} \quad & t \\ \text{satisfying:} \quad & \mathrm{Tr}_{\mathcal{Y}_2}(X) = J(\Phi_1) \\ & \mathrm{Tr}_{\mathcal{Y}_1}(X) = J(\Phi_2) \\ & X \geq t \cdot \mathbb{1}_{\mathcal{X}\otimes\mathcal{Y}_1\otimes\mathcal{Y}_2} \end{aligned} \tag{123}$$

and let β_C be the optimal value of its dual problem, which can be stated as

$$\begin{aligned} \text{minimize:} \quad & \langle Z_1, J(\Phi_1)\rangle + \langle Z_2, J(\Phi_2)\rangle \\ \text{satisfying:} \quad & \mathrm{Tr}^*_{\mathcal{Y}_2}(Z_1) + \mathrm{Tr}^*_{\mathcal{Y}_1}(Z_2) \in \mathrm{D}(\mathcal{X} \otimes \mathcal{Y}_1 \otimes \mathcal{Y}_2) \\ & Z_1 \in \mathrm{Herm}(\mathcal{X} \otimes \mathcal{Y}_1) \\ & Z_2 \in \mathrm{Herm}(\mathcal{X} \otimes \mathcal{Y}_2). \end{aligned} \tag{124}$$

Recall here that $\mathrm{Tr}^*_{\mathcal{Y}_1}$ and $\mathrm{Tr}^*_{\mathcal{Y}_2}$ are the adjoints of the partial trace maps that are defined in Eq. (48).

Strong duality holds for this pair of semidefinite programs, as we now argue. Indeed, the operator

$$\bar{X} = \frac{1}{\dim(\mathcal{Y}_2)} \mathrm{Tr}^*_{\mathcal{Y}_2}\big(J(\Phi_1)\big) + \frac{1}{\dim(\mathcal{Y}_1)} \mathrm{Tr}^*_{\mathcal{Y}_1}\big(J(\Phi_2)\big) - \frac{1}{\dim(\mathcal{Y}_1 \otimes \mathcal{Y}_2)} \mathbb{1}_{\mathcal{X}\otimes\mathcal{Y}_1\otimes\mathcal{Y}_2} \tag{125}$$

is a feasible solution for the primal SDP, and thus

$$\alpha_C \geq \lambda_{\min}(\bar{X}). \tag{126}$$

Similarly, the operators

$$\bar{Z}_1 := \frac{1}{2\dim(\mathcal{X} \otimes \mathcal{Y}_1 \otimes \mathcal{Y}_2)} \mathbb{1}_{\mathcal{X}\otimes\mathcal{Y}_1} \quad \text{and} \quad \bar{Z}_2 := \frac{1}{2\dim(\mathcal{X} \otimes \mathcal{Y}_1 \otimes \mathcal{Y}_2)} \mathbb{1}_{\mathcal{X}\otimes\mathcal{Y}_2} \tag{127}$$

form a strictly feasible dual solution. Thus,

$$\beta_C \leq \frac{1}{\dim(\mathcal{Y}_1 \otimes \mathcal{Y}_2)}. \tag{128}$$

By Slater's theorem (see, e.g., [1, Theorem 1.18]) it holds that $\alpha_C = \beta_C$ and that the optimal value α_C is attained. Summarizing these bounds, we have

$$\lambda_{\min}(\bar{X}) \leq \alpha_C = \beta_C \leq \frac{1}{\dim(\mathcal{Y}_1 \otimes \mathcal{Y}_2)}. \tag{129}$$

Note the fact that α_C is attained tells us something interesting. It tells us that Φ_1 and Φ_2 are compatible if and only if $\alpha_C \geq 0$ (and thus $\beta_C \geq 0$). This brings us to the following theorem.

Theorem 35 (Theorem of the alternative (version 1)). Exactly one of the following statements is true:

(1) $\Phi_1 \in \mathrm{C}(\mathcal{X}, \mathcal{Y}_1)$ and $\Phi_2 \in \mathrm{C}(\mathcal{X}, \mathcal{Y}_2)$ are compatible.

(2) There exists $Z_1 \in \mathrm{Herm}(\mathcal{X} \otimes \mathcal{Y}_1)$ and $Z_2 \in \mathrm{Herm}(\mathcal{X} \otimes \mathcal{Y}_2)$ such that

$$\mathrm{Tr}^*_{\mathcal{Y}_2}(Z_1) + \mathrm{Tr}^*_{\mathcal{Y}_1}(Z_2) \geq 0 \quad \text{and} \quad \langle Z_1, J(\Phi_1)\rangle + \langle Z_2, J(\Phi_2)\rangle < 0. \tag{130}$$

Proof. As stated previously, statement (1) is equivalent to the condition that $\alpha_C \geq 0$. Thus, if statement (1) is not true, then $\alpha_C < 0$ and thus there exists a dual feasible solution $(\bar{Z}_1, \bar{Z}_2)$ with negative objective function value. The pair $(\bar{Z}_1, \bar{Z}_2)$ witnesses that statement (2) is true. In other words, both statements cannot be false.

Now, suppose that both statements are true for the purpose of a contradiction. This implies the existence of a primal feasible solution $\bar{X} \geq 0$ and $Z_1 \in \mathrm{Herm}(\mathcal{X} \otimes \mathcal{Y}_1)$ and $Z_2 \in \mathrm{Herm}(\mathcal{X} \otimes \mathcal{Y}_2)$ such that the conditions in Eq. (130) hold. Now we have

$$\begin{aligned} 0 &> \langle Z_1, J(\Phi_1)\rangle + \langle Z_2, J(\Phi_2)\rangle \\ &= \langle Z_1, \mathrm{Tr}_{\mathcal{Y}_2}(\bar{X})\rangle + \langle Z_2, \mathrm{Tr}_{\mathcal{Y}_1}(\bar{X})\rangle \\ &= \langle \mathrm{Tr}^*_{\mathcal{Y}_2}(Z_1) + \mathrm{Tr}^*_{\mathcal{Y}_1}(Z_2), \bar{X}\rangle \\ &\geq 0, \end{aligned} \tag{131}$$

as each operator is positive semidefinite, which yields a contradiction. Thus, both statements cannot be true. The result follows.

To state a neat corollary, we define an inner product on linear maps.

Definition 36. We may define an inner product on $\mathrm{T}(\mathcal{X}, \mathcal{Y})$ as

$$\langle \Psi, \Phi \rangle := \langle J(\Psi), J(\Phi)\rangle \tag{132}$$

for every choice of linear maps $\Phi, \Psi \in \mathrm{T}(\mathcal{X}, \mathcal{Y})$. That is, the inner product between two linear maps is defined as the inner product of their Choi representations. Note that this is a proper inner product as the Choi representation is an isomorphism.

We now have the following corollary of Theorem 35.

Theorem 37 (Theorem of the alternative (version 2)). Exactly one of the following statements is true:

(1) $\Phi_1 \in \mathrm{C}(\mathcal{X}, \mathcal{Y}_1)$ and $\Phi_2 \in \mathrm{C}(\mathcal{X}, \mathcal{Y}_2)$ are compatible.

(2) There exists Hermitian-preserving maps $\Psi_1 \in \mathrm{T}(\mathcal{X}, \mathcal{Y}_1)$ and $\Psi_2 \in \mathrm{T}(\mathcal{X}, \mathcal{Y}_2)$ such that

$$\mathrm{Tr}^*_{\mathcal{Y}_2} \circ \Psi_1 + \mathrm{Tr}^*_{\mathcal{Y}_1} \circ \Psi_2 \ \text{ is completely positive} \quad \text{and} \quad \langle \Psi_1, \Phi_1\rangle + \langle \Psi_2, \Phi_2\rangle < 0. \tag{133}$$

We now use the machinery we have developed to provide a novel proof of the no-broadcasting theorem in the following example.

Example 38 (No-broadcasting theorem). Recall that the no-broadcasting theorem states that the identity channel is not self-compatible. To study the self-compatibility of the identity channel, we define the partially depolarizing channel $\Omega_p \in \mathrm{C}(\mathcal{X})$ with parameter $p \in [0,1]$, as

$$\Omega_p = p\Omega + (1-p)\mathbb{1}_{\mathrm{L}(\mathcal{X})}. \tag{134}$$

Consider the operators Z_1 and Z_2 defined as

$$Z_1 = Z_2 := \mathbb{1} \otimes \mathbb{1} - \frac{2}{\dim(\mathcal{X})+1} \sum_{i,j=1}^{\dim(\mathcal{X})} E_{i,j} \otimes E_{i,j}. \tag{135}$$

It can be verified that this choice of operators satisfies

$$\mathrm{Tr}^*_{\mathcal{Y}_2}(Z_1) + \mathrm{Tr}^*_{\mathcal{Y}_1}(Z_2) \geq 0 \qquad \text{and} \qquad \langle Z_1 + Z_2, J(\Omega_p) \rangle < 0 \tag{136}$$

for p in the range

$$0 \leq p < \frac{\dim(\mathcal{X})}{2(\dim(\mathcal{X})+1)}. \tag{137}$$

Thus, by Theorem 35, the channel Ω_p is not self-compatible for these values of p (see also [14, 15] for another proof of this fact). Taking $p = 0$ implies the result of the no-broadcasting theorem, as Ω_0 is the identity channel.

Remark 39 (Rewriting the dual using Jordan products). We have shown a few close relationships between the notions of compatibility and Jordan products. We now show that this relationship is rather natural, as the Jordan product appears (in a somewhat hidden form) in the dual SDP above. To see this, observe that

$$\langle Z_1, J(\Phi_1) \rangle + \langle Z_2, J(\Phi_2) \rangle = \langle J(\Phi_1 \odot_A \Phi_2), \mathrm{Tr}^*_{\mathcal{Y}_2}(Z_1) + \mathrm{Tr}^*_{\mathcal{Y}_1}(Z_2) \rangle \tag{138}$$

where $J(\Phi_1 \odot_A \Phi_2)$ is a generalized Jordan product for every choice of operator A satisfying the conditions in Eq. (87). From this we may obtain the following alternative form of the dual problem in Eq. (124):

$$\begin{aligned} \text{minimize:} \quad & \langle J(\Phi_1 \odot_A \Phi_2), \rho \rangle \\ \text{subject to:} \quad & \rho = \mathrm{Tr}^*_{\mathcal{Y}_2}(Z_1) + \mathrm{Tr}^*_{\mathcal{Y}_1}(Z_2) \in \mathrm{D}(\mathcal{X} \otimes \mathcal{Y}_1 \otimes \mathcal{Y}_2) \\ & Z_1 \in \mathrm{Herm}(\mathcal{X} \otimes \mathcal{Y}_1) \\ & Z_2 \in \mathrm{Herm}(\mathcal{X} \otimes \mathcal{Y}_2). \end{aligned} \tag{139}$$

Note that this yields a previous result of ours. Namely, if there exists an operator A satisfying the conditions in Eq. (87) such that $J(\Phi_1 \odot_A \Phi_2) \geq 0$, then from the dual (as written above) we have that $\beta_C \geq 0$ and thus Φ_1 and Φ_2 are compatible.

6.2 SDP for finding a generalized Jordan product compatibilizer

Recall that for channels $\Phi_1 \in \mathrm{C}(\mathcal{X}, \mathcal{Y}_1)$ and $\Phi_2 \in \mathrm{C}(\mathcal{X}, \mathcal{Y}_2)$, the task of determining Jordan compatibility is equivalent to the following problem

$$\begin{aligned} \text{find:} \quad & A \in \mathrm{Herm}(\mathcal{X} \otimes \mathcal{X}_1 \otimes \mathcal{X}_2) \\ \text{satisfying:} \quad & \mathrm{Tr}_{\mathcal{X}_1}(A) = J(\mathbb{1}_{\mathrm{L}(\mathcal{X})}) \\ & \mathrm{Tr}_{\mathcal{X}_2}(A) = J(\mathbb{1}_{\mathrm{L}(\mathcal{X})}) \\ & (\mathbb{1}_{\mathrm{L}(\mathcal{X})} \otimes \Phi_1 \otimes \Phi_2)(A) \in \mathrm{Pos}(\mathcal{X} \otimes \mathcal{Y}_1 \otimes \mathcal{Y}_2) \end{aligned} \tag{140}$$

where $J(\Phi_1 \odot_A \Phi_2) = \big(\mathbb{1}_{\mathrm{L}(\mathcal{X})} \otimes \Phi_1 \otimes \Phi_2\big)(A)$ is the generalized Jordan product of Φ_1 and Φ_2 with respect to A.

The problem of determining Jordan compatibility of a pair of channels can be phrased in terms of the following pair of semidefinite programs. Let α_{J} be the optimal value of the following semidefinite program

$$\begin{aligned} \text{maximize:} \quad & t \\ \text{satisfying:} \quad & \mathrm{Tr}_{\mathcal{X}_1}(A) = J(\mathbb{1}_{\mathrm{L}(\mathcal{X})}) \\ & \mathrm{Tr}_{\mathcal{X}_2}(A) = J(\mathbb{1}_{\mathrm{L}(\mathcal{X})}) \\ & \big(\mathbb{1}_{\mathrm{L}(\mathcal{X})} \otimes \Phi_1 \otimes \Phi_2\big)(A) \geq t \cdot \mathbb{1}_{\mathcal{X}\otimes\mathcal{Y}_1\otimes\mathcal{Y}_2} \\ & A \in \mathrm{Herm}(\mathcal{X} \otimes \mathcal{X}_1 \otimes \mathcal{X}_2), \end{aligned} \tag{141}$$

and let β_{J} be the optimal value of the corresponding dual problem, which can be stated as follows

$$\begin{aligned} \text{minimize:} \quad & \langle W_1 + W_2, J(\mathbb{1}_{\mathrm{L}(\mathcal{X})})\rangle \\ \text{satisfying:} \quad & (\mathbb{1}_{\mathrm{L}(\mathcal{X})} \otimes \Phi_1^* \otimes \Phi_2^*)(\rho) = \mathrm{Tr}^*_{\mathcal{X}_2}(W_1) + \mathrm{Tr}^*_{\mathcal{X}_1}(W_2) \\ & W_1 \in \mathrm{Herm}(\mathcal{X} \otimes \mathcal{X}_1) \\ & W_2 \in \mathrm{Herm}(\mathcal{X} \otimes \mathcal{X}_2) \\ & \rho \in \mathrm{D}(\mathcal{X} \otimes \mathcal{Y}_1 \otimes \mathcal{Y}_2). \end{aligned} \tag{142}$$

We now show that strong duality holds for this pair of semidefinite programs. To see this, note that the operator $A = A_{\mathrm{JP}}$ together with the value $t = \lambda_{\min}(\big(\mathbb{1}_{\mathrm{L}(\mathcal{X})} \otimes \Phi_1 \otimes \Phi_2\big)(A_{\mathrm{JP}}))$ forms a feasible solution to the primal problem in Eq. (141), and thus

$$\alpha_{\mathrm{J}} \geq \lambda_{\min}\Big(\big(\mathbb{1}_{\mathrm{L}(\mathcal{X})} \otimes \Phi_1 \otimes \Phi_2\big)(A_{\mathrm{JP}})\Big). \tag{143}$$

Similarly, the operators

$$W_1 := \frac{1}{2\dim(\mathcal{X})^3}\,\mathbb{1}_{\mathcal{X}\otimes\mathcal{X}_1} \tag{144}$$

$$W_2 := \frac{1}{2\dim(\mathcal{X})^3}\,\mathbb{1}_{\mathcal{X}\otimes\mathcal{X}_2} \tag{145}$$

$$\rho := \frac{1}{\dim(\mathcal{X})^3}\,\mathbb{1}_{\mathcal{X}\otimes\mathcal{Y}_1\otimes\mathcal{Y}_2} \tag{146}$$

form a strictly feasible solution to the dual problem, and thus

$$\beta_{\mathrm{J}} \leq \frac{1}{\dim(\mathcal{X})^2}. \tag{147}$$

Strong duality now follows from the fact that the primal is feasible and the dual is strictly feasible. Moreover, it holds that $\alpha_{\mathrm{J}} = \beta_{\mathrm{J}}$ and that the optimal value α_{J} is attained. Summarizing these bounds, we have

$$\lambda_{\min}\Big(\big(\mathbb{1}_{\mathrm{L}(\mathcal{X})} \otimes \Phi_1 \otimes \Phi_2\big)(A_{\mathrm{JP}})\Big) \leq \alpha_{\mathrm{J}} = \beta_{\mathrm{J}} \leq \frac{1}{\dim(\mathcal{X})^2}. \tag{148}$$

Again, note that the fact that $\alpha_{\rm J}$ is attained tells us something interesting. It tells us that Φ_1 and Φ_2 are Jordan compatible if and only if $\alpha_{\rm J} \geq 0$ (and thus $\beta_{\rm J} \geq 0$). This brings us to the following theorem.

Theorem 40 (Theorem of the alternative (Jordan version)). Exactly one of the following statements is true:

(1) $\Phi_1 \in \mathrm{C}(\mathcal{X}, \mathcal{Y}_1)$ and $\Phi_2 \in \mathrm{C}(\mathcal{X}, \mathcal{Y}_2)$ are Jordan compatible.

(2) There exists $W_1 \in \mathrm{Herm}(\mathcal{X} \otimes \mathcal{X}_1)$, $W_2 \in \mathrm{Herm}(\mathcal{X} \otimes \mathcal{X}_2)$, and $\rho \in \mathrm{Pos}(\mathcal{X} \otimes \mathcal{Y}_1 \otimes \mathcal{Y}_2)$ such that

$$(\mathbb{1}_{\mathrm{L}(\mathcal{X})} \otimes \Phi_1^* \otimes \Phi_2^*)(\rho) = \mathrm{Tr}^*_{\mathcal{X}_2}(W_1) + \mathrm{Tr}^*_{\mathcal{X}_1}(W_2) \quad \text{and} \quad \langle W_1 + W_2, J(\mathbb{1}_{\mathrm{L}(\mathcal{X})}) \rangle < 0. \tag{149}$$

Proof. The proof is completely analogous to that of Theorem 35.

Remark 41 (Rewriting this dual using Jordan products). Again, we can use Jordan products to rewrite the dual SDP in Eq. (142). In particular, the dual objective function can be given by any of the four equivalent expressions:

$$\begin{aligned} \langle W_1 + W_2, J(\mathbb{1}_{\mathrm{L}(\mathcal{X})}) \rangle &= \langle A, \mathrm{Tr}^*_{\mathcal{X}_2}(W_1) + \mathrm{Tr}^*_{\mathcal{X}_1}(W_2) \rangle \\ &= \langle A, (\mathbb{1} \otimes \Phi_1^* \otimes \Phi_2^*)(\rho) \rangle \\ &= \langle J(\Phi_1 \odot_A \Phi_2), \rho \rangle \end{aligned} \tag{150}$$

where A satisfies the conditions in Eq. (87). Note that they are equivalent since we are assuming (W_1, W_2, ρ) is dual feasible.

Supplementary Note 7 - Qubit channels

We now consider the case where $\mathcal{X} = \mathcal{Y} = \mathbb{C}^2$. From a result about the symmetric extendibility of two-qubit states [16], we have that a qubit channel $\Phi \in \mathrm{C}(\mathcal{X})$ is self-compatible if and only if it holds that

$$\mathrm{Tr}\left(\left(\mathrm{Tr}_{\mathcal{X}}(J(\Phi))\right)^2 \right) \geq \mathrm{Tr}(J(\Phi)^2) - 4\sqrt{\det(J(\Phi))}. \tag{151}$$

However, closed-form algebraic criteria for the compatibility of other channels remain unknown.

7.1 Self-compatibility of dephasing-depolarizing channels

In this subsection, we consider the compatibility and k-self-compatibility of a class of channels that we call dephasing-depolarizing channels, which we define as follows. For fixed parameters $p, q \in [0,1]$ satisfying $p + q \leq 1$, the partially dephasing-depolarizing channel is the linear map

$$\Xi_{p,q} = (1 - p - q)\mathbb{1}_{\mathrm{L}(\mathcal{X})} + p\Delta + q\Omega, \tag{152}$$

where we recall the (completely) dephasing channel Δ and the (completely) depolarizing channel Ω given as

$$\Delta(X) = \sum_{i=1}^{\dim(\mathcal{X})} \mathrm{Tr}(E_{i,i}X)E_{i,i}, \qquad \text{and} \qquad \Omega(X) = \frac{\mathrm{Tr}(X)}{\dim(\mathcal{X})}\mathbb{1}_{\mathcal{X}}. \tag{153}$$

It can be easily verified that $\Xi_{p,q}$ is a measure-and-prepare channel (i.e., its Choi representation is separable, or in this case, PPT) if and only if

$$\frac{2(1-p)}{3} \leq q \leq 1-p. \tag{154}$$

(which is easy to check from the PPT criterion). Thus, for values of p and q in this region, the channel $\Xi_{p,q}$ is k-self-compatible for all k. We now consider the k-self-compatibility for these channels for small values of k.

The case $k = 2$ is simple, as one may apply Eq. (151) to determine self-compatibility. Note that $\mathrm{Tr}_{\mathcal{X}}(J(\Xi_{p,q})) = \mathbb{1}_{\mathcal{Y}}$ and thus $\mathrm{Tr}((\mathrm{Tr}_{\mathcal{X}}(J(\Xi_{p,q})))^2) = 2$ holds for these channels. It is then straightforward to verify that Eq. (151) is satisfied for $p, q \in [0,1]$ with $p + q \leq 1$ if and only if

$$\frac{2-p-\sqrt{1+2p(1-p)}}{3} \leq q \leq 1-p. \tag{155}$$

This yields the region for which $\Xi_{p,q}$ is self-compatible. The lower bound of this region determined by the inequality in Eq. (155) is part of the ellipse defined by the equation

$$p^2 + 2pq + 3q^2 - 2p - 4q + 1 = 0. \tag{156}$$

Note that if one could not obtain a closed-form expression for the self-compatibility of a given family of channels analytically, it could be computed numerically by solving the relevant SDPs.

For $k \in \{3, \ldots 10\}$, we also numerically determine the regions where $\Xi_{p,q}$ is k-self-compatible using the SDP solver CVX [17, 18]. The results are depicted in Figure 1. Note that for $k \geq l$, k-self-compatibility implies l-self-compatibility. (We note that although we did not give the SDP explicitly for k-self-compatibility, one can extend the SDP for self-compatibility in the natural way. In fact, it is equivalent to the k-symmetric extendibility of the (normalized) Choi representation, and the SDP is then given in [19].)

Interestingly, it appears that the regions of k-self-compatibility agree with the regions determined by the inequality

$$f_k(p) \leq q \leq 1-p \tag{157}$$

where f_k is the function defined by

$$f_k(p) = \begin{cases} \frac{2}{3}\left(\frac{k-1}{k} - p\right), & 0 \leq p \leq \frac{(k-2)(k-1)}{k(k+1)} \\ \frac{k-1}{k^2-k+1}\left(p + k(1-p) - \sqrt{k-1+p(2-kp)}\right), & \frac{(k-2)(k-1)}{k(k+1)} < p \leq 1. \end{cases} \tag{158}$$

For $k = 2$ this agrees with Eq. (155). The curve defined by the points satisfying $f_k(p) = q$ (i.e. the lower boundary of this region) for $\frac{(k-2)(k-1)}{k(k+1)} < p \leq 1$ is part of the ellipse defined by the equation

$$p^2 + 2pq + \frac{k^2-k+1}{(k-1)^2}q^2 - 2p - \frac{2k}{k-1}q + 1 = 0. \tag{159}$$

The boundaries of the regions defined by the inequalities in Eq. (157) are depicted as the purple curves in Figure 1.

Let us not look at the Jordan self-compatibility of $\Xi_{p,q}$. Note that, for parameters $p, q > 0$ satisfying $p + q < 1$, the map $\Xi_{p,q}$ is invertible as a linear map. Indeed, the inverse map is given as

$$\Xi_{p,q}^{-1} = \frac{1}{1-p-q}\left(\mathbb{1}_{\mathrm{L}(\mathcal{X})} - \frac{p}{1-q}\Delta - q\frac{1-p-q}{1-q}\Omega\right). \tag{160}$$

By Proposition 24, and for these values of p and q, the channel $\Xi_{p,q}$ is therefore self-compatible if and only if it is (generalized) Jordan compatible with itself. Recall that every measure-and-prepare channel is self-compatible. Thus, the region of pairs (p, q) such that $\Xi_{p,q}$ is a measure-and-prepare channel (i.e., such that the Choi representation $J(\Xi_{p,q})$ is a separable operator) forms a subset of the region of pairs for which $\Xi_{p,q}$ is self-compatible. Consider now the standard Jordan product of $\Xi_{p,q}$ with itself. If the map $\Xi_{p,q} \odot \Xi_{p,q}$ is completely positive (i.e., if $J(\Xi_{p,q} \odot \Xi_{p,q}) \geq 0$), then $\Xi_{p,q}$ is self-compatible. It is not difficult (using Mathematica) to analytically compute the spectrum of the Choi representation of $\Xi_{p,q} \odot \Xi_{p,q}$. In fact, it holds that $J(\Xi_{p,q} \odot \Xi_{p,q}) \geq 0$ if and only if

$$\frac{3-2p-\sqrt{3-2p^2}}{3} \leq q \leq 1-p. \tag{161}$$

The lower bound of this region determined by the inequality in Eq. (161) is part of the ellipse defined by the equation

$$p^2 + 2pq + \frac{3}{2}q^2 - 2p - 3q + 1 = 0. \tag{162}$$

The boundaries of these three regions are depicted in Figure 1. Interestingly, the region where $J(\Xi_{p,q} \odot \Xi_{p,q}) \geq 0$ is strictly smaller than the region where $\Xi_{p,q}$ is self-compatible and strictly larger than the region where $J(\Xi_{p,q})$ is separable.

7.2 Compatibility of depolarizing channels

We now restrict our attention to pairs of partially depolarizing channels $(\Omega_{q_0}, \Omega_{q_1})$ as defined in Eq. (134). (To connect this analysis to the discussion in the previous section, note channels of this form may be expressed as $\Omega_q = \Xi_{0,q}$.) It is evident that $\Omega = \Omega_1$ is compatible with every channel (see Lemma 18). Moreover it is known that the channel Ω_q is self-compatible whenever $1/3 \leq q \leq 1$ (see [14] for details). Furthermore, the set of pairs of compatible channels must be convex. Hence, the region of pairs (q_0, q_1) for which Ω_{q_0} and Ω_{q_1} are compatible must at least contain the convex hull of these pairs. In fact, it was shown in [7] that the channels $(\Omega_{q_0}, \Omega_{q_1})$ are compatible if and only if

$$q_0 + \sqrt{q_0 q_1} + q_1 \geq 1. \tag{163}$$

We add to this analysis by considering pairs (q_0, q_1) where $J(\Omega_{q_0} \odot \Omega_{q_1}) \geq 0$. (That is, when Ω_{q_0} and Ω_{q_1} are (standard) Jordan compatible.) One can easily determine the region where the standard Jordan product of Ω_{q_0} and Ω_{q_1} is completely positive. Computing the spectrum of the Choi representation $J(\Omega_{q_0} \odot \Omega_{q_1})$ using Mathematica, one finds these channels are Jordan product compatible if and only if

$$3q_0q_1(4-q_0)(4-q_1) + 12(1-q_0-q_1)^2 - 40q_0q_1 \leq 0. \tag{164}$$

To illustrate the distinction between these three different regions of compatibility, the boundaries of these regions are depicted in Figure 2. These are the boundaries of the regions of values (q_0, q_1) where

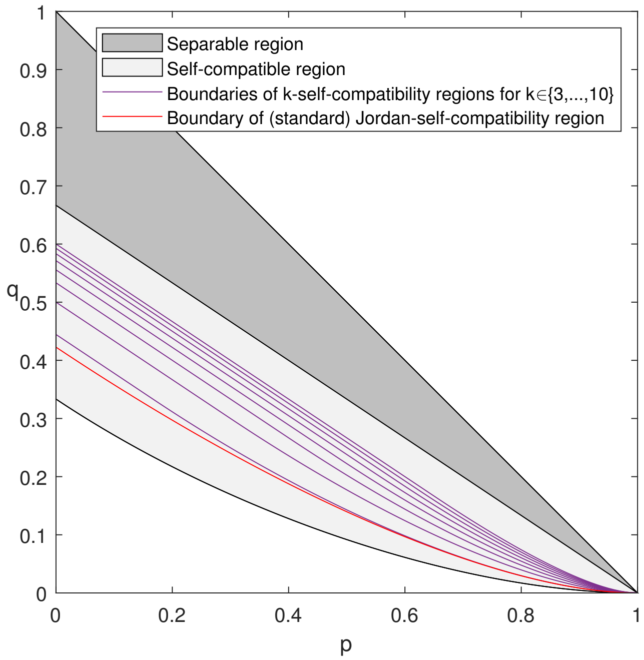

Supplementary Figure 1: The regions of k-self-compatibility and (standard) Jordan self-compatibility of the partially dephasing-depolarizing channel. The topmost (black) line is $p + q = 1$ below which is the region in consideration. The next black line down is the lower boundary of the region given by Eq. (154) where $\Xi_{p,q}$ is k-self-compatible for all values of k. The bottom-most curve (black) is the lower boundary of the region given by Eq. (155) where $\Xi_{p,q}$ is 2-self-compatible. The next curve (red) is the lower boundary of the region given by Eq. (161) where $J(\Xi_{p,q} \odot \Xi_{p,q}) \geq 0$, i.e., where $\Xi_{p,q}$ is (standard) Jordan self-compatible. The subsequent curves above represent the boundaries regions where $\Xi_{p,q}$ is k-self-compatible channels for $k \in \{3, \ldots, 10\}$ which were determined numerically (using CVX).

- Ω_{q_0} and Ω_{q_1} are compatible (black curve),
- $J(\Omega_{q_0} \odot \Omega_{q_1}) \geq 0$ (i.e., where Ω_{q_0} and Ω_{q_1} are (standard) Jordan compatible) (red curve), and
- $(\Omega_{q_0}, \Omega_{q_1})$ is in the convex hull of the self-compatible pair $(\Omega_{1/3}, \Omega_{1/3})$ and the trivially compatible pairs (Ω_0, Ω_1), (Ω_1, Ω_0), and (Ω_1, Ω_1) (blue curve)

(where we note that every channel is trivially compatible with the completely depolarizing channel $\Omega_1 = \Omega$). Interestingly, the region of points (q_0, q_1) satisfying $J(\Omega_{q_0} \odot \Omega_{q_1}) \geq 0$ is neither a subset nor a superset of the convex hull of the points $(1/3, 1/3)$, $(0, 1)$, $(1, 0)$, and $(1, 1)$. This illustrates that the (standard) Jordan product provides an interesting non-trivial sufficient condition for the compatibility of these channels.

Note that for values of q satisfying $0 \leq q < 1$, the partially depolarizing map Ω_q is invertible

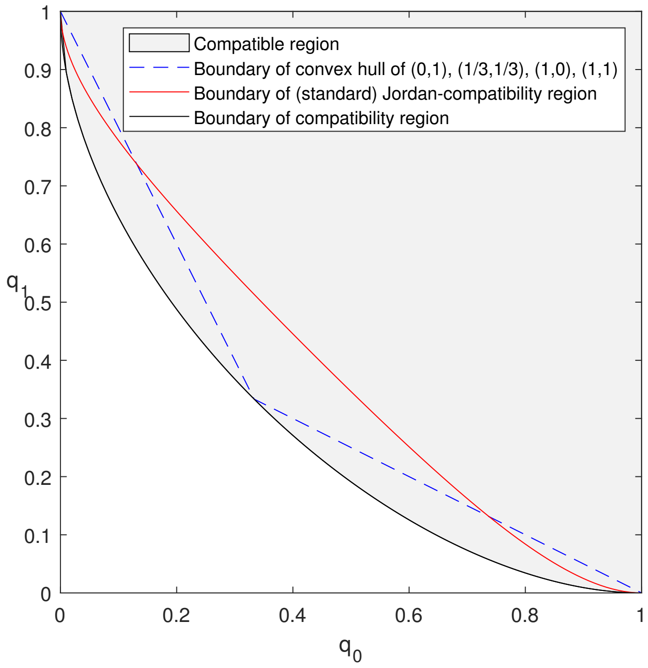

Supplementary Figure 2: Regions of points (q_0, q_1) where the channels Ω_{q_0} and Ω_{q_1} satisfy each given property. The black curve is the lower boundary of the region where the channels Ω_{q_0} and Ω_{q_1} are compatible. The red curve is the lower boundary of the region where $J(\Omega_{q_0} \odot \Omega_{q_1}) \geq 0$. The blue curve is the lower boundary of the convex hull of the points $(1/3, 1/3)$, $(0,1)$, $(1,0)$, and $(1,1)$.

with inverse map given by

$$\Omega_q^{-1} = \frac{1}{1-q}\left(\mathbb{1}_{\mathrm{L}(\mathcal{X})} - q\Omega\right). \tag{165}$$

Therefore, for parameters satisfying $0 < q_0 < 1$ and $0 < q_1 < 1$, the channels Ω_{q_0} and Ω_{q_1} are compatible if and only if they are (generalized) Jordan compatible.

References

[1] Watrous, J. *The Theory of Quantum Information* (Cambridge University Press, 2018).

[2] Heinosaari, T., Miyadera, T. & Ziman, M. An invitation to quantum incompatibility. *Journal of Physics A: Mathematical and Theoretical* **49**, 123001 (2016).

[3] Heinosaari, T. & Miyadera, T. Incompatibility of quantum channels. *Journal of Physics A: Mathematical and Theoretical* **50**, 135302 (2017).

[4] Plávala, M. Conditions for the compatibility of channels in general probabilistic theory and their connection to steering and Bell nonlocality. *Physical Review A* **96**, 052127 (2017).

[5] Kuramochi, Y. Quantum incompatibility of channels with general outcome operator algebras. *Journal of Mathematical Physics* **59**, 042203 (2018).

[6] Kuramochi, Y. Entanglement-breaking channels with general outcome operator algebras. *Journal of Mathematical Physics* **59**, 102206 (2018).

[7] Haapasalo, E. Compatibility of Covariant Quantum Channels with Emphasis on Weyl Symmetry. *Annales Henri Poincaré* **20**, 3163–3195 (2019).

[8] Haapasalo, E., Kraft, T., Miklin, N. & Uola, R. Quantum marginal problem and incompatibility Preprint at https://arxiv.org/abs/1909.02941 (2019).

[9] Heinosaari, T., Leppäjärvi, L. & Plávala, M. No-free-information principle in general probabilistic theories. *Quantum* **3**, 157 (2019).

[10] Horodecki, M., Horodecki, P. & Horodecki, R. Separability of mixed states: necessary and sufficient conditions. *Physics Letters A* **223**, 1–8 (1996).

[11] Heinosaari, T. A simple sufficient condition for the coexistence of quantum effects. *Journal of Physics A: Mathematical and Theoretical* **46** (2013).

[12] Heinosaari, T., Reitzner, D. & Stano, P. Notes on joint measurability of quantum observables. *Foundations of Physics* **38**, 1133–1147 (2008).

[13] Lerner, N. *A Course on Integration Theory* (Springer, 2014).

[14] Werner, R. F. Optimal cloning of pure states. *Physical Review A* **58**, 1827–1832 (1998).

[15] Keyl, M. & Werner, R. F. Optimal cloning of pure states, testing single clones. *Journal of Mathematical Physics* **40**, 3283–3299 (1999).

[16] Chen, J., Ji, Z., Kribs, D., Lütkenhaus, N. & Zeng, B. Symmetric extension of two-qubit states. *Physical Review A* **90**, 032318 (2014).

[17] Grant, M. & Boyd, S. CVX: Matlab software for disciplined convex programming, version 2.1. http://cvxr.com/cvx (2014).

[18] Grant, M. & Boyd, S. Graph implementations for nonsmooth convex programs. In Blondel, V., Boyd, S. & Kimura, H. (eds.) *Recent Advances in Learning and Control*, Lecture Notes in Control and Information Sciences, 95–110 (Springer-Verlag Limited, 2008). http://stanford.edu/~boyd/graph_dcp.html.

[19] Doherty, A. C., Parrilo, P. A. & Spedalieri, F. M. Complete family of separability criteria. *Physical Review A* **69**, 022308 (2004).
